# Identification of KRAS mutation-associated gut microbiota in colorectal cancer and construction of predictive machine learning model

**DOI:** 10.1128/spectrum.02720-23

**Published:** 2024-04-04

**Authors:** Zigui Huang, Xiaoliang Huang, Yili Huang, Kunmei Liang, Lei Chen, Chuzhuo Zhong, Yingxin Chen, Chuanbin Chen, Zhen Wang, Fuhai He, Mingjian Qin, Chenyan Long, Binzhe Tang, Yongqi Huang, Yongzhi Wu, Xianwei Mo, Tang Weizhong, Jungang Liu

**Affiliations:** 1 Division of Colorectal & Anal Surgery, Department of Gastrointestinal Surgery, Guangxi Medical University Cancer Hospital, Nanning, China; 2 College of Oncology, Guangxi Medical University, Nanning, China; Central Texas Veterans Health Care System, Temple, Texas, USA

**Keywords:** colorectal cancer, KRAS mutation, 16S rRNA, gut microbiota, machine learning

## Abstract

Gut microbiota has demonstrated an increasingly important role in the onset and development of colorectal cancer (CRC). Nonetheless, the association between gut microbiota and KRAS mutation in CRC remains enigmatic. We conducted 16S rRNA sequencing on stool samples from 94 CRC patients and employed the linear discriminant analysis effect size algorithm to identify distinct gut microbiota between KRAS mutant and KRAS wild-type CRC patients. Transcriptome sequencing data from nine CRC patients were transformed into a matrix of immune infiltrating cells, which was then utilized to explore KRAS mutation-associated biological functions, including Gene Ontology items and Kyoto Encyclopedia of Genes and Genomes pathways. Subsequently, we analyzed the correlations among these KRAS mutation-associated gut microbiota, host immunity, and KRAS mutation-associated biological functions. At last, we developed a predictive random forest (RF) machine learning model to predict the KRAS mutation status in CRC patients, based on the gut microbiota associated with KRAS mutation. We identified a total of 26 differential gut microbiota between both groups. Intriguingly, a significant positive correlation was observed between *Bifidobacterium* spp. and mast cells, as well as between *Bifidobacterium longum* and chemokine receptor CX3CR1. Additionally, we also observed a notable negative correlation between *Bifidobacterium* and GOMF:proteasome binding. The RF model constructed using the KRAS mutation-associated gut microbiota demonstrated qualified efficacy in predicting the KRAS phenotype in CRC. Our study ascertained the presence of 26 KRAS mutation-associated gut microbiota in CRC and speculated that *Bifidobacterium* may exert an essential role in preventing CRC progression, which appeared to correlate with the upregulation of mast cells and CX3CR1 expression, as well as the downregulation of GOMF:proteasome binding. Furthermore, the RF model constructed on the basis of KRAS mutation-associated gut microbiota exhibited substantial potential in predicting KRAS mutation status in CRC patients.

**IMPORTANCE:**

Gut microbiota has emerged as an essential player in the onset and development of colorectal cancer (CRC). However, the relationship between gut microbiota and KRAS mutation in CRC remains elusive. Our study not only identified a total of 26 gut microbiota associated with KRAS mutation in CRC but also unveiled their significant correlations with tumor-infiltrating immune cells, immune-related genes, and biological pathways (Gene Ontology items and Kyoto Encyclopedia of Genes and Genomes pathways). We speculated that *Bifidobacterium* may play a crucial role in impeding CRC progression, potentially linked to the upregulation of mast cells and CX3CR1 expression, as well as the downregulation of GOMF:Proteasome binding. Furthermore, based on the KRAS mutation-associated gut microbiota, the RF model exhibited promising potential in the prediction of KRAS mutation status for CRC patients. Overall, the findings of our study offered fresh insights into microbiological research and clinical prediction of KRAS mutation status for CRC patients.

## INTRODUCTION

Colorectal cancer (CRC) stands as a prevalent common gastrointestinal malignancy, ranking third worldwide in terms of cancer incidence and second in terms of mortality in 2020 (1). The majority of CRC cases arise in economically developed regions (2), and recent years have witnessed a continuous year-on-year increase in the global incidence of CRC. Some experts projected a 63% increase in new cases and a 73% rise in CRC-related deaths each year (3), posing a great threat to human life and health.

There exists a multitude of causes for CRC, such as an unhealthy lifestyle, an unbalanced dietary structure, and a family history of CRC (4). Multiple studies have substantiated the growing significance of the gut microbiota as a key factor closely intertwined with the onset of CRC. The gut microbiota is a complex microecosystem within the human body, comprised of various types of microorganisms that interact and exert mutual constraints within the intestines, playing a vital role in various physiological processes (5). The human gut microbiota consists of a vast array of microorganisms, with more than 10^10^ types of bacteria and a population that can range from 10^13^ to 10^14^ in a normal human body (6). The role of gut microbiota in CRC is two-sided, contributing to either the development and progression of CRC or the suppression of tumor activity (7). Studies have demonstrated that enterotoxigenic *Bacteroides fragilis* can induce inflammatory diarrhea and the development of CRC through the secretion of *Bacteroides fragilis* toxin (8). Inversely, *Lactobacillus reuteri* strains can secrete chemicals such as short-chain fatty acids, thereby reducing the risk of CRC occurrence. Imbalances in the intestinal flora are frequently observed in CRC patients (9). Consequently, improving the structure of gut microbiota aims to enhance the proportion of beneficial bacteria in the intestinal flora, effectively diminishing the development of CRC (10).

Furthermore, KRAS gene mutation has emerged as a pivotal factor in CRC development and progression (11). The KRAS gene serves as a significant oncogene in CRC, with around 40% of CRC patients experiencing KRAS gene mutation (12). In the case of cells with an abnormal KRAS mutation status, they experience continuous stimulation for growth, ultimately leading to the emergence and development of tumors (12). Previous studies have illustrated that KRAS mutation activates the epidermal growth factor receptor signaling pathway, resulting in the persistent activation of the KRAS protein and disrupting the normal cell growth pattern while promoting abnormal cell proliferation (11). In addition, several lines of evidence support the notion that gut microbiota is also associated with the abnormal proliferation of tumor cells. For instance, *Fusobacterium nucleatum* has proved its contribution to the proliferation and development of CRC cell lines by regulating the TLR4/MYD88/NF-κB pathway and upregulating the miR-21 gene expression (13). Nonetheless, in the realm of CRC research, little is known about the linkage between gut microbiota and KRAS mutation simultaneously.

With the rapid development of biological technology, the era of biological big data has dawned (14). Bioinformatics not only aids in comprehending the mechanisms behind cancer occurrence and development but also introduces a novel approach to the early diagnosis, prevention, and treatment of cancer (15, 16). mRNA sequencing (RNA-Seq) assumes a significant role in the domain of bioinformatics, as it enables the precise measurement of gene transcription status and the quantification of gene expression (17, 18). Through bioinformatics analysis of mRNA sequencing data, the molecular underpinnings of cancer initiation and development can be elucidated from multiple perspectives (19–21).

In short, our study aimed to identify differential gut microbiota between KRAS mutant and KRAS wild-type CRC patients by fecal 16S rRNA gene sequencing. In the next place, we intended to analyze their correlations with immune infiltrating cells, immune-related genes, and enriched biological functions, all of which will be derived from the analysis of mRNA sequencing data, aiming to demystify their roles in KRAS mutation and the progression of CRC. Moreover, we will employ these KRAS mutation-associated gut microbiota to construct a predictive machine learning model, offering fresh insights into microbiological discovery and clinical prediction of KRAS mutation status for CRC patients.

## MATERIALS AND METHODS

### Collection of samples

After the research proposal received approval from the Medical Ethics Committee of the Guangxi Medical University Cancer Hospital, and with the consent of the patients, we collected stool samples from 236 CRC patients admitted to the Guangxi Medical University Cancer Hospital during the period from 1 January 2021 to 31 December 2021. Among these, 198 stool samples met the qualifications for 16S rRNA sequencing. Following the initial hospital admission, we obtained demographic and clinicopathological data, including gender, age, TNM stage, KRAS phenotype, lymph-vascular invasion, and perineural invasion, from the medical records of these 198 patients. In this study, we analyzed 94 stool samples containing information on KRAS mutation status, concluding 24 KRAS mutant cases and 70 KRAS wild-type cases (for five samples without KRAS mutation status information, we predicted their status using consistent clustering analysis based on corresponding transcriptome sequencing data). Additionally, we collected nine tumor samples from the above patients after surgery for transcriptome sequencing.

The inclusion criteria for the sample collection were as follows: (i) CRC patients who had undergone surgical treatment and had clear pathological staging according to the American Joint Council on Cancer 8th Edition CRC staging guidelines (22) or patients diagnosed with colorectal adenocarcinoma through a colonoscopy pathological biopsy, (ii) CRC patients with no history of other malignant tumors, (iii) CRC patients with no concurrent intestinal diseases or acute complications such as intestinal perforation, complete intestinal obstruction, or pelvic abscess, (iv) CRC patients who had not received any prior antitumor therapy (such as surgery, chemotherapy, radiotherapy, immunotherapy, and traditional Chinese medicine therapy) before stool sample collection, (v) CRC patients with no antibiotics or intestinal microbioecologics administered within 1 month prior to enrollment (23, 24), and (vi) CRC patients with no history of consciousness injury or other cognitive dysfunction.

We collected fecal samples from the patients on the first day after their admission and provided instructions to retain the middle portion of the feces while avoiding urine contamination when using a sterile fecal collection tube. The collected fecal specimens were then divided into 2 mL EP tubes (200 mg/tube) and stored in sterile ice boxes at −80°C for freezing storage. The surgically removed fresh tumor tissue samples were soybean-sized (about 3 to 5 mm in diameter) and stored in liquid nitrogen within 30 minutes after removal from surgery.

### 16S RNA sequencing of fecal samples and gut microbiota analysis

The quality of DNA in the stool sample was assessed before sequencing. Two hundred milligrams of fecal sample was added to Tris-EDTA buffer for DNA extraction, utilizing the MOBIO PowerSoil DNA Isolation Kit. Following DNA quality analysis, DNA extraction was carried out, and only high-quality DNA was subjected to PCR amplification. With 341F (5′-CCTACGGGNGGCWGCAG-3′) and 805R (5′-GACTACHVGGGTATCTAATCC-3) as primers (25), the V3 and V4 regions of the 16S rRNA gene were targeted for capture and selectively amplified by PCR. Post-PCR amplification, the PCR products were assessed via a 2% agarose-gel electrophoresis assay, targeting bands within the range of 300 to 350 bp. Quantitative analysis of the PCR products was performed using the Quant-iT PicoGreen dsDNA Assay Kit. After equal molar concentration pooling of results from all samples, the combined samples were quantitatively analyzed using the KAPA Library Quantification Kit KK4824. Finally, qualified hybrid libraries were sequenced using a 2 × 250 bp chemical reagent on the Illumina PE250 apparatus.

Following the retrieval of FASTQ raw sequencing data for each sample, we filtrated the sequencing data by Quantitative Insights Into Microbial Ecology version 2 (QIIME2) to obtain high-quality raw sequencing data. Species of gut microbiota were annotated with the Greengene database v13.8, followed by amplicon sequence variant(ASV)/operational taxonomic unit(OTU) extraction of gut microbiota in the “phyloseq” package v1.26.1. The diversity of gut microbiota in each sample was assessed using α diversity indices, where Chao1 and ACE indices described community richness, while Shannon index and Simpson index characterized community diversity and evenness. β diversity was employed to depict the compositional diversity within each sample within the same ecological environment. This was carried out through ADONIS and ANOSIM analyses using the “vegan” package v2.5.6. Additionally, partial least squares-discriminant analysis (PLS-DA) was conducted using the “mixOmics” v6.6.2 package, which is suitable for high-dimensional data classification and comparison. Linear discriminant analysis effect size (LEfSe) was used to filter gut microbiota that are likely to explain variations between different populations (26). In our study, linear discriminant analysis (LDA) was performed to screen differential gut microbiota between groups with LEfSe software v1.0.0 under the threshold of |LDA score| > 2 and *P*-value < 0.05. Following a logarithmic transformation with base 10, the LDA score was employed to assess the impact of differential gut microbiota, visualized in the form of a bar chart using the “ggplot2” package v3.4.0. For the selected gut microbiota, the larger the absolute value of the LDA score, the more effectively they can distinguish between KRAS mutant and KRAS wild-type CRC patients. Finally, we used Phylogenetic investigations of Communities by Reconstruction of Unobserved States II (PICRUSt2) software 2.3.0 to predict the enrichment of Kyoto Encyclopedia of Genes and Genomes (KEGG) pathways between KRAS mutant and KRAS wild-type CRC patients based on 16S rRNA sequencing data. A non-parametric Mann–Whitney test was used to compare α and β diversity indices and enriched KEGG pathways between both groups through the “vegan” package v2.5.6. All these procedures were executed in R software v3.5.1. All statistical tests were two-tailed, and statistical significance was only determined for *P*-value < 0.05.

### Transcriptome sequencing of tumor tissue samples

Total RNA was extracted from nine CRC tumor samples using the TRIzol total RNA extraction kit, successively proceeded with RNA integrity detection by electrophoresis and RNA purity determination by a microultraviolet spectrophotometer. Subsequently, stranded RNA was eliminated, and cDNA libraries were constructed using the VAHTS Stranded mRNA-Seq Library Preparation Kit for Illumina. The resulting transcriptome library underwent sequencing on the Illumina NovaSeq 6000 platform. Then, FastQC was used to evaluate the quality of the raw data obtained by sequencing. In short, HISAT2 (version: hg38) was utilized to align the sample’s valid data to the Genome Reference Consortium Human Build 38 Organism (GRCh38). StringTie and known gene models were employed for assessing gene expression, with the transcripts per million (TPM) calculated to represent the abundance of each gene’s expression.

### Consensus clustering analysis

Nine patients with CRC underwent fecal 16S rRNA sequencing and tumor transcriptome sequencing, with four of them having a confirmed KRAS mutation status (two cases in the KRAS mutant group and two cases in the KRAS wild-type group). Differentially expressed genes (DEGs) between the KRAS mutant and KRAS wild-type groups were identified using the “edgeR” package (version 3.40.0) with the criteria of |log2 fold change (log2FC)| > 1 and false discovery rate (FDR) < 0.05. Unsupervised learning is a machine learning approach used to discover hidden structures and patterns within unlabeled data. Consensus clustering (CC) is an unsupervised learning technique that aggregates multiple weak bases into more stable clusters through random partition methods (27). Utilizing the DEGs expression matrix of the nine samples, the nine CRC patients were categorized into two groups using the CC method to predict the KRAS mutation status of the remaining five samples, employing the “ConsensusClusterPlus” package (version 1.62.0) with a specific cluster count of *k* = 2. Principal component analysis (PCA) was employed to extract the most informative features from a reduced set of principal components, simplifying data complexity while retaining underlying trends and patterns (28). Utilizing the “FactoMine” package (version 2.8) and the “factoextra” R package (version 1.0.7), PCA was conducted on the DEGs expression matrix related to KRAS mutation status in the nine samples to validate the predictions obtained through the CC method. Ultimately, the KRAS mutation status of the remaining five samples was determined, and these samples were included in the study for further analysis.

### Immune infiltration analysis of CRC

CIBERSORT is a gene expression algorithm designed to detect the presence of immune cells within the tumor microenvironment based on the unique gene expression tags of each tumor-infiltrating immune cell (TIIC) (29). This algorithm is applicable to any genomic mixture that adheres to its mathematical model (30). In our study, we employed the CIBERSORT R script version 1.03 to analyze immune infiltration in nine CRC patients with tumor transcriptome sequencing data. We converted their TPM matrices into a relative content matrix for 22 different immune cell types by constructing the feature matrix based on the microarray data from the CIBERSORT algorithm.

### Functional enrichment analysis of KRAS mutation associated with transcriptome sequencing

The single sample gene set enrichment analysis (ssGSEA) algorithm sequences the enriched genes in each marker gene set by descending order of expression level and calculates the ssGSEA score for each gene set (31). Each ssGSEA enrichment score represents the extent to which members of a particular gene set in a sample are coordinately upregulated or downregulated, which is applicable for the analysis of RNA sequencing data (32) and for the detection of enriched immune cell infiltration and biological functional pathways within each sample (33). We downloaded the required gene sets in GMT format (c2.cp.kegg.v2022.1.Hs.symbols.gmt, c5.go.v2022.1.Hs.symbols. gmt) from the GSEA website (https://www.gsea-msigdb.org/gsea/index.jsp). In the “GSVA” package v1.46.0, we utilized the “ssgsea” algorithm to calculate the gene set scoring matrix for each sample. Subsequently, the KRAS wild-type group was designated as the control group. We applied the “limma” algorithm within the “TCGAbiolinks” package v2.25.3 to analyze differential Gene Ontology (GO) items and KEGG pathways between the two groups, using a significance threshold of *P*-value < 0.05 and |log2FC| > 0. The GO analysis encompassed three aspects: Biological Process (BP), Molecular Function (MF), and Cellular Component (CC). For the visualization of the screening results of GO items and KEGG pathways, we employed the “ggplot2” package v3.4.0 to create a volcano plot. The above operations were performed in R software v4.2.2.

### Construction of machine learning model and identification of intestinal microbial markers

In order to predict KRAS mutation status in CRC patients, we developed a random forest (RF) model to identify intestinal microbial markers. The RF model is a machine learning algorithm based on a decision tree foundation (34), whose primary advantage lies in its ability to optimize all variables using non-linear risk functions without making any necessary assumptions (35). Moreover, RF is particularly well suited for modeling outcomes based on genomic data, effectively harnessing numerous features with weak information, even in the absence of explicit data (36). To begin with, we randomly divided a cohort of 94 CRC patients into training and validation sets in a 7:3 ratio. We initiated the process by employing the sklearn.feature_selection model to evaluate the significance of KRAS mutation-associated gut microbiota, which subsequently served as the features for constructing the RF model. These features were then scored, with higher scores indicating greater importance. Subsequently, we ranked all the KRAS mutation-associated gut microbiota according to their importance scores; we set an appropriate proportion of features to be further included in the construction of the RF model. The RF model based on gut microbiota was established using default parameters. We installed the necessary software packages for building and evaluating the RF machine learning model on the Python and SciKit Learn 0.18 platforms (https://scikit-learn.org/stable/). The accuracy of the RF model was evaluated separately on the training and validation sets, using the area under the curve (AUC) of the receiver operating characteristic (ROC).

### Statistical methods

Using R software v4.2.2 for data analysis, we specified that all *P*-values were double-tailed, and *P*-value < 0.05 was deemed statistically significant. The *t*-test and Pearson chi-squared test were applied for continuous and categorical variables, respectively (Table 1), in SPSS v23.0. We calculated the correlations between dominant bacteria in different groups, immune cells, and immune-related genes using Pearson correlation. Additionally, Spearman correlation was used to determine the relationships between dominant bacteria in different groups. These calculations were carried out using the “Hmisc” package version 4.7-1. Spearman correlation was also utilized to calculate the correlation coefficient between dominant bacteria of different groups and enriched BP items, MF items, and KEGG pathways with “ggstatsplot” package v0.10.0. The Benjamini–Hochberg method was used as the method for multiple comparison correction, which was implemented via the p.adjust function of “ggcorrplot” package v4.2.2, where the method argument was set to “BH.” The correlation heat maps were generated with the assistance of the “pheatmap” package version 1.0.8 and the “ggcorrplot” package version 0.1.4. For visualizing correlation matrices in the form of a correlation network graph, we employed the “Igraph” package version 1.3.5 and the Cytoscape software version 3.7.2.

**TABLE 1:** Demographic and clinical characteristics of CRC patients stratified by KRAS mutation status^*a*^

		KRAS mutant CRC patients (*n* = 24)	KRAS wild-type CRC patients (*n* = 70)	*P*-value	Test
Age [years, mean (SD)]		56.13 ± 13.04	58.54 ± 11.14	0.382	*t*-test
Age (%)	≥60	9 (37.50)	30 (42.90)	0.646	Pearson chi square
	<60	15 (62.50)	40 (57.10)		
Gender (%)	Male	12 (50.00)	44 (62.90)	0.268	Pearson chi square
	Female	12 (50.00)	26 (37.10)		
TNM stage (%)	Early (0–2)	19 (79.20)	42 (60.90)	0.104	Pearson chi square
	Advanced (3–4)	5 (20.80)	27 (39.10)		
Lymph-vascular invasion (%)	Yes	8 (36.40)	27 (42.90)	0.594	Pearson chi square
	No	14 (63.60)	36 (57.10)		
Perineural invasion (%)	Yes	9 (42.90)	38 (60.30)	0.163	Pearson chi square
	No	12 (57.10)	25 (39.70)		

^*a*^ There was statistical significance only when the *P-*value was <0.05.

## RESULTS

### General information and clinical characteristics of recruited CRC patients

**Fig 1:**
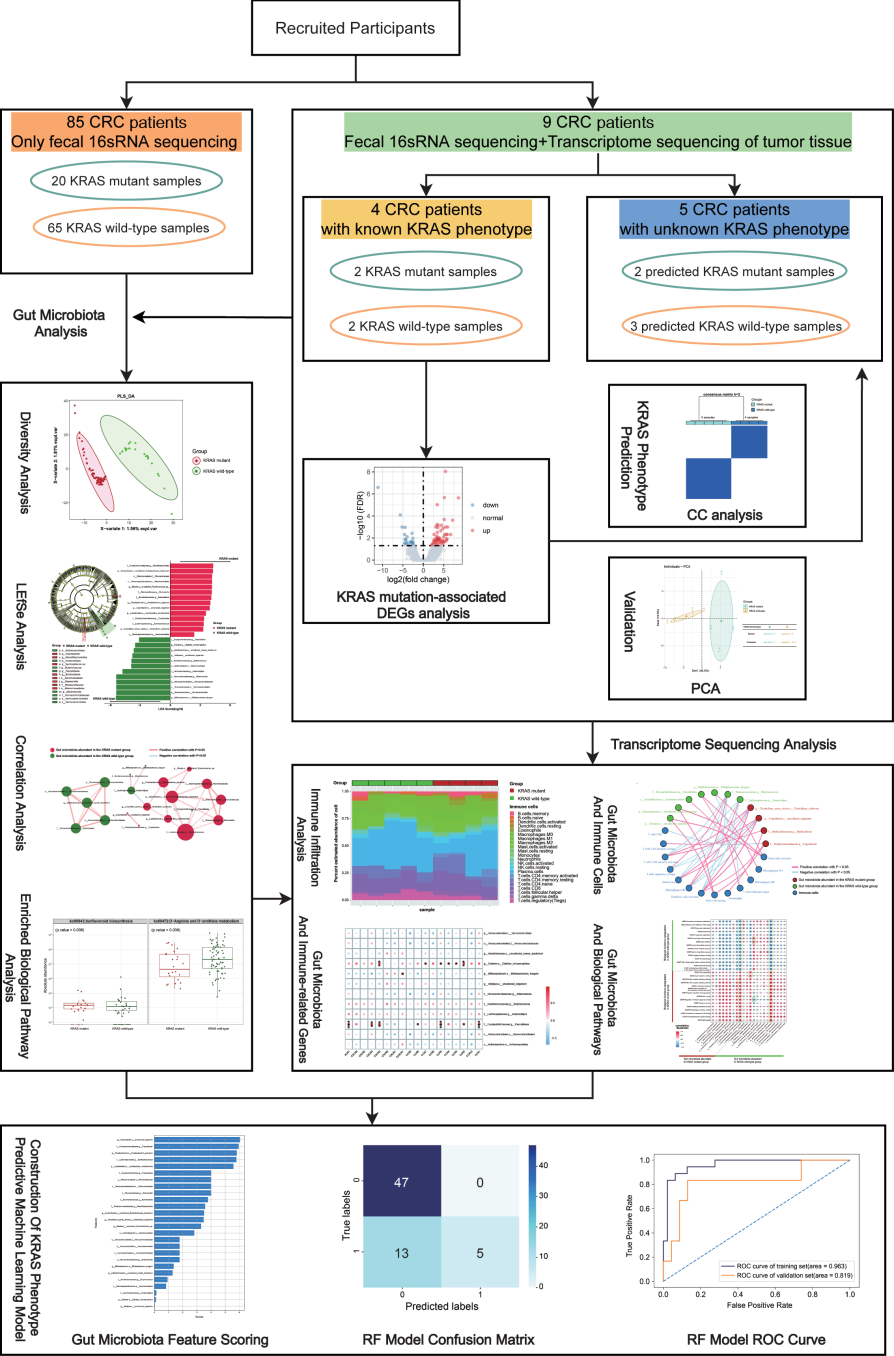
Flow chart of this research. The information of participants, main structure, and analysis methods of this study are shown.

A total of 94 CRC patients were included in this study (the KRAS mutation status of five samples was predicted by the CC approach), as detailed in Table 1. The information on recruited participants, major analytical methods, and the main structure of this study are shown in the flow chart (Fig. 1). This cohort comprised 24 KRAS mutant and 70 KRAS wild-type CRC patients, respectively. There were no statistically significant differences in terms of age and sex, suggesting that the baseline data in our study were well balanced and comparable. Furthermore, no significant statistical relationships were observed in TNM staging, lymph-vascular invasion, and perineural invasion between both groups (*P* > 0.05).

To make full use of all the nine samples with transcriptome sequencing for further bioinformatics analysis, we performed DEGs analysis on four CRC samples with both KRAS phenotype and transcriptome sequencing data to obtain KRAS mutation-related DEGs and utilized the CC approach to predict the KRAS mutation status in another five CRC patients on the basis of the KRAS mutation-related DEGs expression matrix of these nine samples. According to the KRAS phenotype, four CRC patients with transcriptome sequencing data were divided into the KRAS mutant group and the KRAS wild-type group to identify KRAS mutation-related DEGs, and we found that there were 77 KRAS mutation-related DEGs, among which 55 DGEs in the KRAS mutant group were significantly upregulated and 22 DEGs were significantly downregulated compared with the KRAS wild-type group, as shown in Fig. S1A and Table S1. Through the CC approach, the nine CRC patients were divided into two subgroups, comprising five cases in subgroup 1 and four cases in subgroup 2; notably, subgroup 1 housed two cases of KRAS wild-type CRC patients, whereas subgroup 2 accommodated two cases of KRAS mutant CRC patients, as illustrated in Fig. S1B. PCA was subsequently applied to validate the clustering groups obtained through consistent clustering. As demonstrated in Fig. S1C, it became apparent that the nine CRC patients could be stratified into two distinct clusters. Specifically, samples 4 and 5 represented KRAS wild-type CRC patients, and samples 6 and 8 were indicative of KRAS mutant CRC patients. The result of PCA further reinforced the differentiation of the nine samples into two subgroups. Leveraging the predicted clustering information derived from consistent clustering, we ascertained the predictive KRAS mutation labels for the remaining five patients and incorporated them into further analysis. Specifically, samples 3 and 7 were projected to be KRAS mutant, while samples 1, 2, and 9 were projected to be KRAS wild-type.

### Comparison of diversity between gut microbiota of KRAS mutant and KRAS wild-type CRC patients

**Fig 2:**
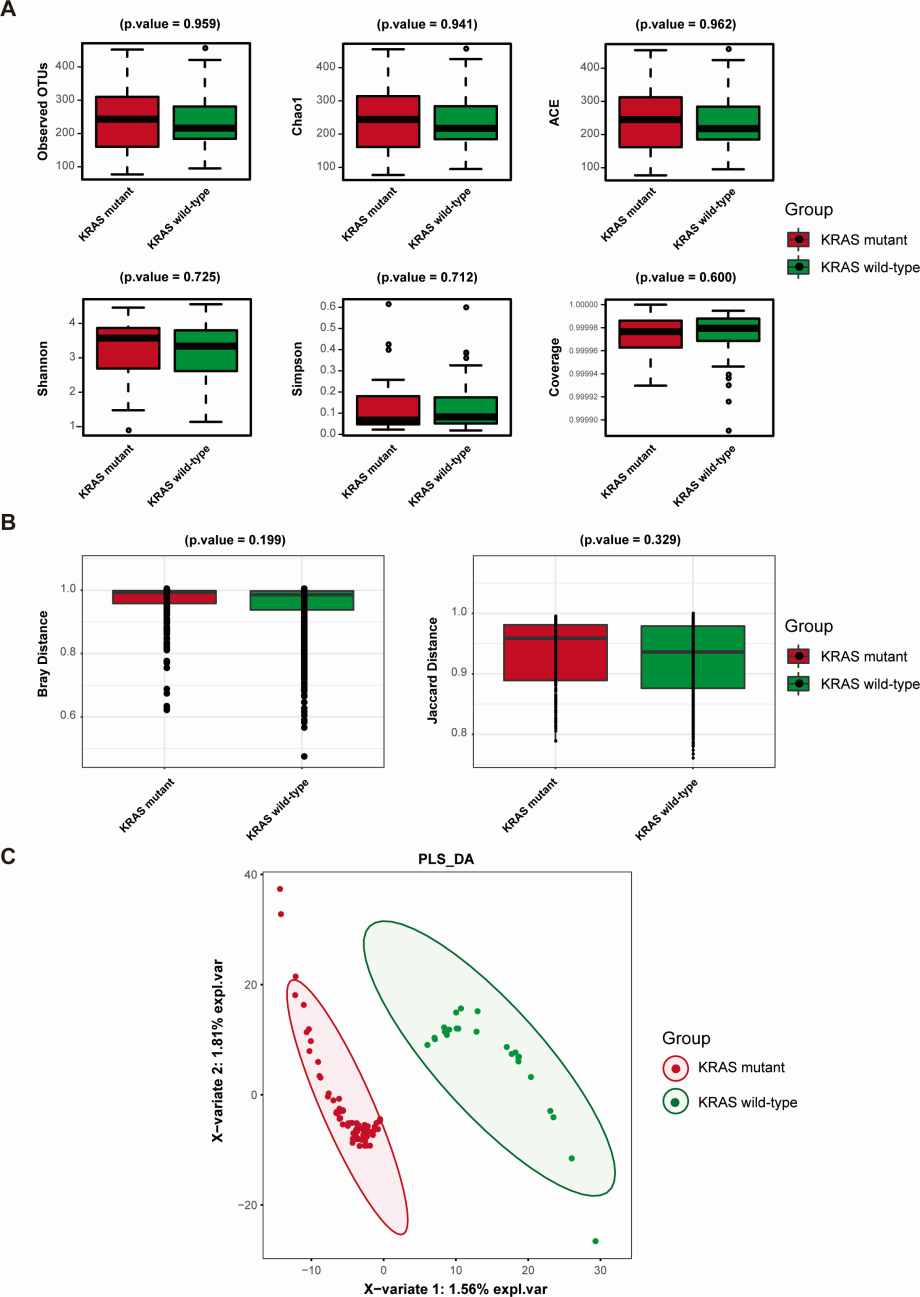
Comparison of gut microbiota diversity indices between the KRAS mutant CRC group and KRAS wild-type CRC group. (**A**) Comparison of α diversity indices between the gut microbiota of the KRAS mutant CRC group and KRAS wild-type CRC group. (**B**) Comparison of β diversity indices of gut microbiota between the KRAS mutant CRC group and KRAS wild-type CRC group. The horizontal axis represents the group, while the vertical axis indicates the community diversity index value for each group of samples. (**C**) PLS-DA of gut microbiota of CRC patients within the KRAS mutant group and KRAS wild-type group. Each dot represents a sample, with colors indicating the respective group. The scales on the horizontal and vertical axes represent the relative distances of each sample. *X*-variable 1 and *X*-variable 2 denote the factors influencing the changes in gut microbiota composition in CRC patients within the KRAS mutant and KRAS wild-type CRC groups.

To begin with, after calculation for six α diversity indices of gut microbiota between KRAS mutant and KRAS wild-type groups, we observed that the observed OTU index, Chao1 index, ACE index, and Shannon index of the KRAS mutant group were higher than those of the KRAS wild-type group, while Simpson index was lower than that of the KRAS wild-type group, albeit without statistical significance (as depicted in Fig. 2A, *P* > 0.05). Subsequently, an evaluation of Bray and Jaccard distance within the gut microbiota revealed that the two indices in the KRAS mutant group surpassed those in the KRAS wild-type group, yet statistical significance remained absent (Fig. 2B, *P* > 0.05). Nevertheless, in the context of PLS-DA, a clear differentiation between KRAS mutant and wild-type CRC patients was evident, as illustrated in Fig. 2C. In summary, while statistical disparities in the richness and diversity of gut microbiota were not observed, discernible variations in microbial composition persisted between both groups.

### Identification of gut microbiota associated with KRAS mutation in CRC

**Fig 3:**
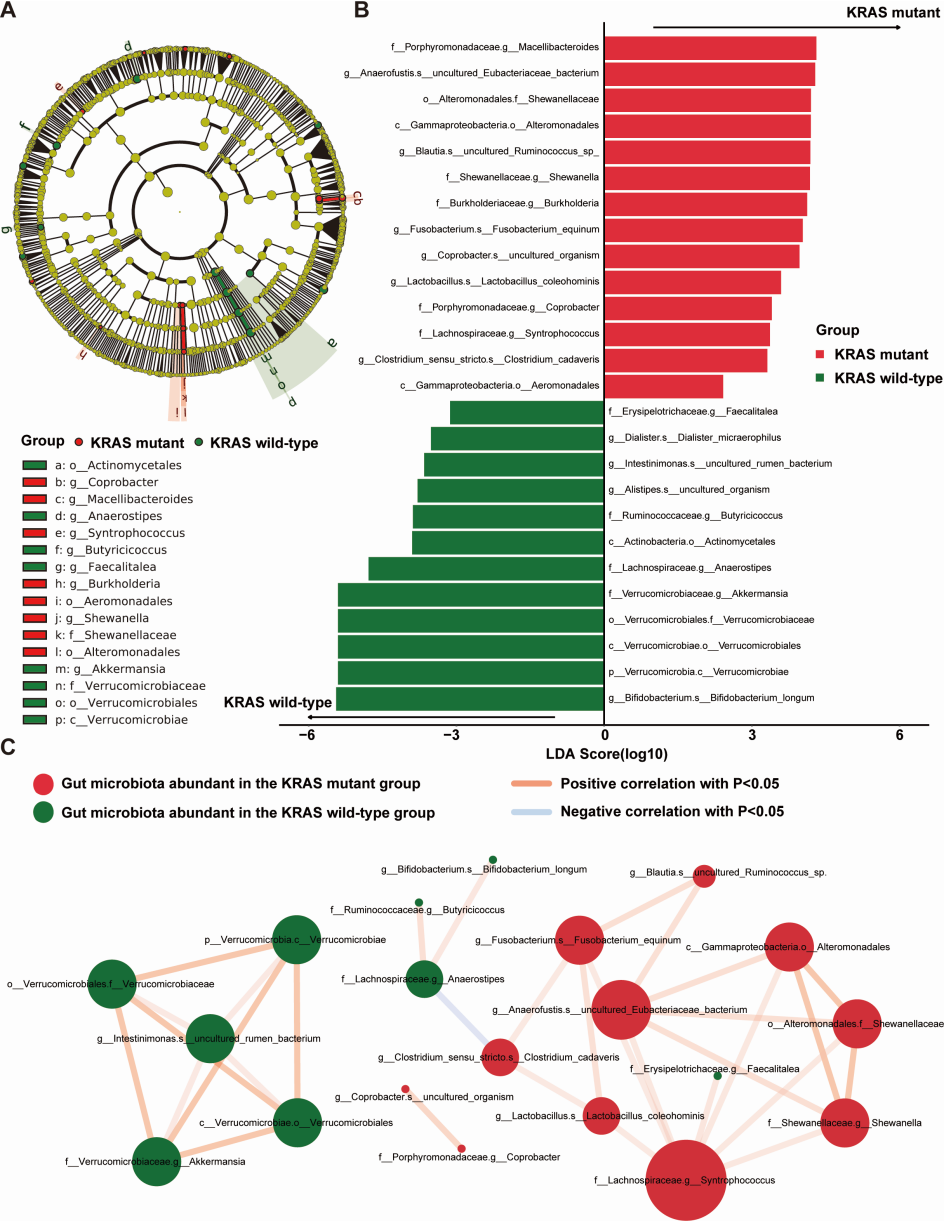
Differential analysis of gut microbiota between the KRAS mutant CRC group and KRAS wild-type CRC group. (**A**) Cladogram of LEfSe analysis. Nodes of various colors signify the categorization of gut microbiota. Yellow nodes indicate species that exhibit no significant differences between the two groups, red nodes signify species with significantly higher abundance in the KRAS mutant group, and green nodes denote species with significantly higher abundance in the KRAS wild-type group. The node’s size corresponds to the abundance of the species; larger nodes indicate higher species abundance. The circle illustrates the taxonomic hierarchy, with the innermost layer representing phylum, followed by class, order, family, genus, and species. Species marker notations within each hierarchy are organized from outer to inner. (**B**) LDA bar graph on the basis of 16S rRNA sequencing. The LDA scores after log10 treatment are plotted as the horizontal coordinates; the color of the histogram indicates the analysis of species with significantly higher abundance in each group, with red denoting the KRAS mutant group and green denoting the KRAS wild-type group. The length of each bar reflects the magnitude of the LDA score value. (**C**) Network diagram of KRAS mutation-associated gut microbiota correlations. Node represents a distinct species, with node color signifying the grouping. Node size correlates positively with the number of connected edges; larger nodes indicate a greater number of connected edges. The presence of a connecting line signifies a significant correlation between two nodes. Blue lines represent a negative Spearman correlation coefficient, while positive Spearman correlation coefficients are depicted in orange lines. The thickness of the line corresponds to the magnitude of the Spearman correlation coefficient between the two nodes.

To identify dominant gut microbiota as biomarkers, LEfSe analysis was performed for both KRAS mutant and KRAS wild-type CRC patients. A total of 26 bacteria with statistically significant differences in abundance were identified. Among these, 14 bacteria exhibited significantly higher abundance in the KRAS mutant group compared to the KRAS wild-type group, while 12 bacteria showed significantly higher abundance in the KRAS wild-type group in comparison to the KRAS mutant group (*P*-value < 0.05, as illustrated in Table S2; Fig. 3A and B). Figure 3B portrays the LDA bar plot, presenting the LDA scores (post-log10 transformation) from the LEfSe analysis for each of the significant bacteria. Furthermore, we constructed a correlation network diagram to explore the interactions among the dominant flora of both groups, specifically focusing on gut microbiota associated with KRAS mutation (Fig. 3C). *f__Lachnospiraceae.g__Syntrophococcus*, *g__Anaerofustis.s__uncultured_Eubacteriaceae_bacteriu,* and *f__Verrucomicrobiaceae.g__Akkermansia* were the three bacteria genus and bacteria species most closely associated with other dominant gut microbiota. There were significant positive correlations observed among the majority of dominant gut microbiota in both groups. Notably, *f__Lachnospiraceae.g__Anaerostipes* in the KRAS wild-type group exhibited a significant negative correlation with *g__Clostridium_sensu_stricto.s__Clostridium_cadaveris* in the KRAS mutant group. To sum up, the correlation network analysis of gut microbiota associated with KRAS mutation indicated different microbial interactions.

### Prediction of biological function between gut microbiota of KRAS mutant and KRAS wild-type CRC patients

**Fig 4:**
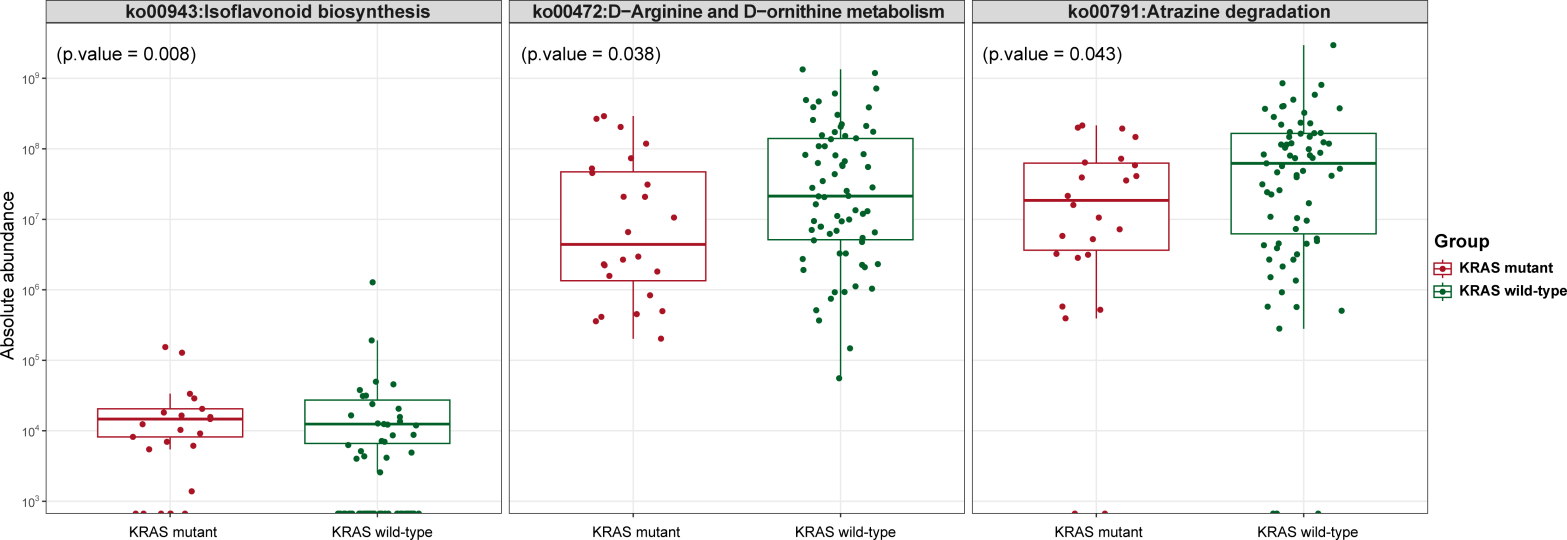
Box plot of KEGG functional abundance between the KRAS mutant CRC group and KRAS wild-type CRC group. The horizontal axis represents the grouping, while the vertical axis represents the predicted abundance. Within each sample, the box’s edges signify the 25th to 75th percentile range, the horizontal black line represents the median, and the extended lines correspond to 1.5 times the maximum and minimum values within the interquartile range.

To investigate the biological pathway enriched in gut microbiota associated with KRAS mutation, we used PICRUSt2 software to screen for differential KEGG pathways between the KRAS mutant and KRAS wild-type groups. A total of 172 KEGG pathways were identified, with only three of them exhibiting statistically significant differences (*P* < 0.05). Among these, two KEGG pathways were upregulated in the KRAS mutant group, displaying greater abundance compared to the KRAS wild-type group. These pathways included D-arginine and D-ornithine metabolism (*P* = 0.038) and atrazine degradation (*P* = 0.043). Conversely, only one KEGG pathway, isoflavonoid biosynthesis (*P* = 0.008), was upregulated in the KRAS wild-type group, and its abundance was significantly higher than that of the KRAS mutant group (as illustrated in Fig. 4 and Table S3, *P*-value < 0.05). These findings indicated distinct metabolic functions within the gut microbiota associated with KRAS mutation.

### Relationship between KRAS mutation-associated gut microbiota and tumor-infiltrating immune cells

**Fig 5:**
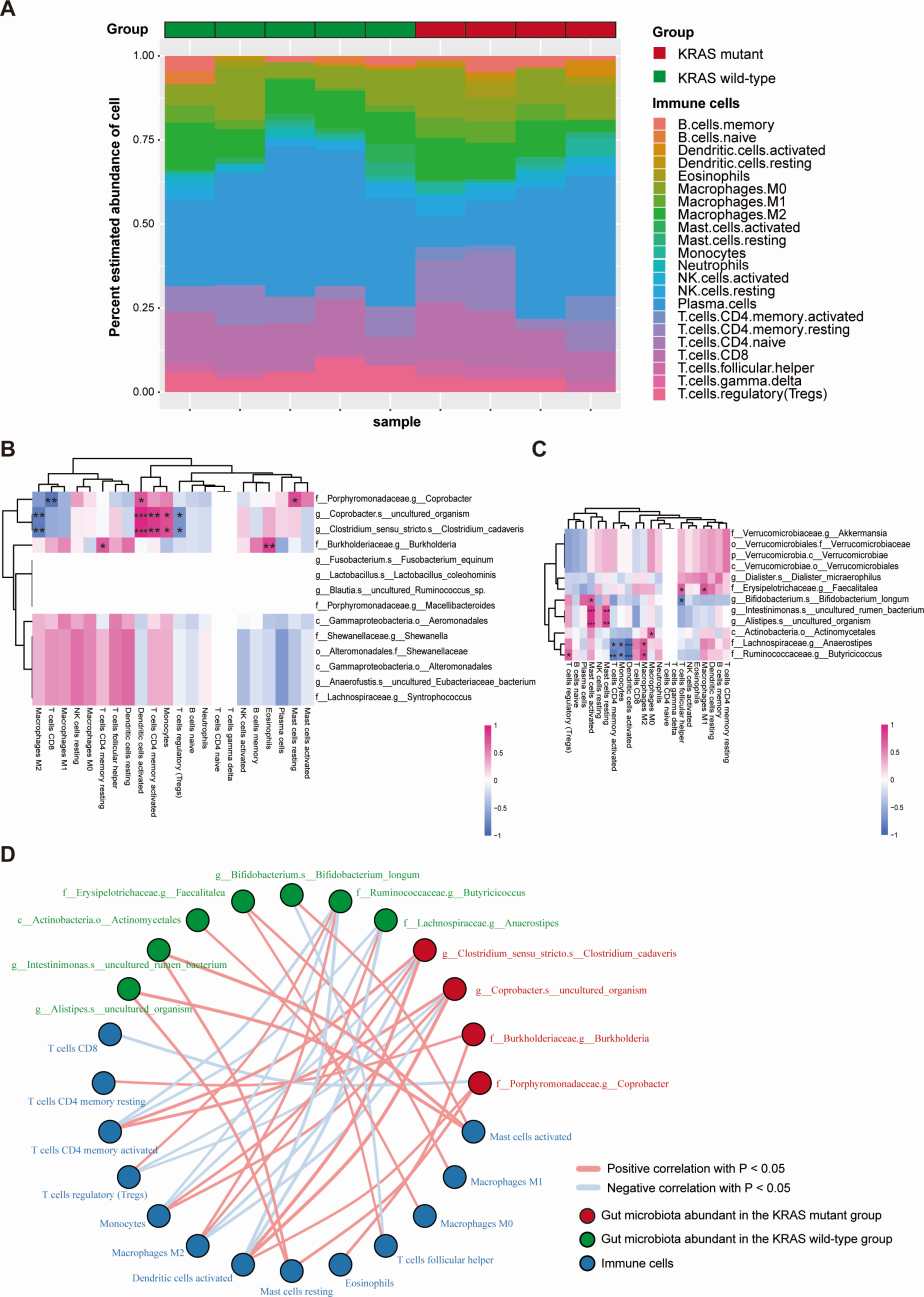
Relationship between KRAS mutation-associated gut microbiota and infiltrating immune cells in CRC. (**A**) Bar graph of relative abundance of infiltrating immune cells in CRC samples. Nine CRC patients were stratified into two groups: the KRAS mutant group and the KRAS wild-type group. Each bar in the figure represents a sample, with each color denoting a specific type of immune cell. The vertical axis displays the relative abundance of infiltrating immune cells, and the sum of these values within a single sample equals 1. (**B**) Heat map of correlation between the dominant gut microbiota in the KRAS mutant group and tumor-infiltrating immune cells. (**C**) Heat map of correlation between dominant gut microbiota in the KRAS wild-type group and tumor-infiltrating immune cells. The horizontal axis represents immune cells, and the vertical axis signifies gut microbiota. Red indicates a positive correlation, while blue indicates a negative correlation. The color intensity reflects the size of the Pearson correlation coefficient. A gradient from light to dark represents a correlation coefficient value from small to large. Additionally, the presence of asterisks in the graph indicates the significance of the *P*-value: no asterisk implies a *P*-value greater than or equal to 0.05, one asterisk represents a *P*-value ranging from 0.01 to less than 0.05, two asterisks indicate a *P*-value from 0.001 to less than 0.01, and three asterisks denote a *P*-value less than 0.001. (**D**) Network diagram of correlation between the KRAS mutation-associated gut microbiota and tumor-infiltrating immune cells. The diagram differentiates between gut microbiota and immune cells with distinct colored nodes. Orange nodes represent gut microbiota, while blue nodes represent immune cells. Connecting lines between nodes signify significant correlations. Blue lines indicate a negative correlation with a Pearson correlation coefficient value less than 0, while red lines indicate a positive correlation with Pearson correlation coefficient values greater than 0. *P*-value < 0.05 in the graph indicates the statistical significance of the displayed data.

TIICs play a pivotal role in the constitution of tumors. They are intricately involved in the antitumor immune response and hold promise as potential drug targets for clinical tumor diagnosis and treatment. To delve into the relationship between KRAS mutation-associated gut microbiota and TIICs, we initiated our study by constructing a bar graph illustrating the composition of 22 distinct types of TIICs in a cohort of nine CRC patients (refer to Fig. 5A), which effectively showcased the distinctive characteristics of the tumor microenvironment unique to each individual of CRC patients. Subsequently, we explored the connection between KRAS mutation-associated gut microbiota and TIICs by scrutinizing the correlations among the predominant gut microbiota in both the KRAS mutant and KRAS wild-type groups and the 22 types of TIICs in a sequential manner. In the KRAS mutant group, we observed that *g__Coprobacter.s__uncultured_organism* and *g__Clostridium_sensu_stricto.s__Clostridium_cadaverism* exhibited a significant positive correlation with activated dendritic cells and activated CD4^+^T memory cells. Conversely, *f__Porphyromonadaceae.g__Coprobacter* demonstrated a notable negative correlation with CD8^+^T cells (refer to Fig. 5B and D). In the KRAS wild-type group, *f__Erysipelotrichaceae.g__Faecalitalea* displayed a significant positive correlation with M1 macrophages and follicular helper T cells. Furthermore, *f__Lachnospiraceae.g__Anaerostipes* and *f__Ruminococcaceae.g__Butyricicoccus* were notably negatively correlated with activated dendritic cells (Fig. 5C and D). In summary, our findings revealed discernible disparities in the composition of TIICs between CRC patients in the KRAS mutant group and those in the KRAS wild-type group. Additionally, several KRAS mutation-associated gut microbiota demonstrated significant correlations with various TIIC subsets.

### Correlation between KRAS mutation-associated gut microbiota and immune-related genes

**Fig 6:**
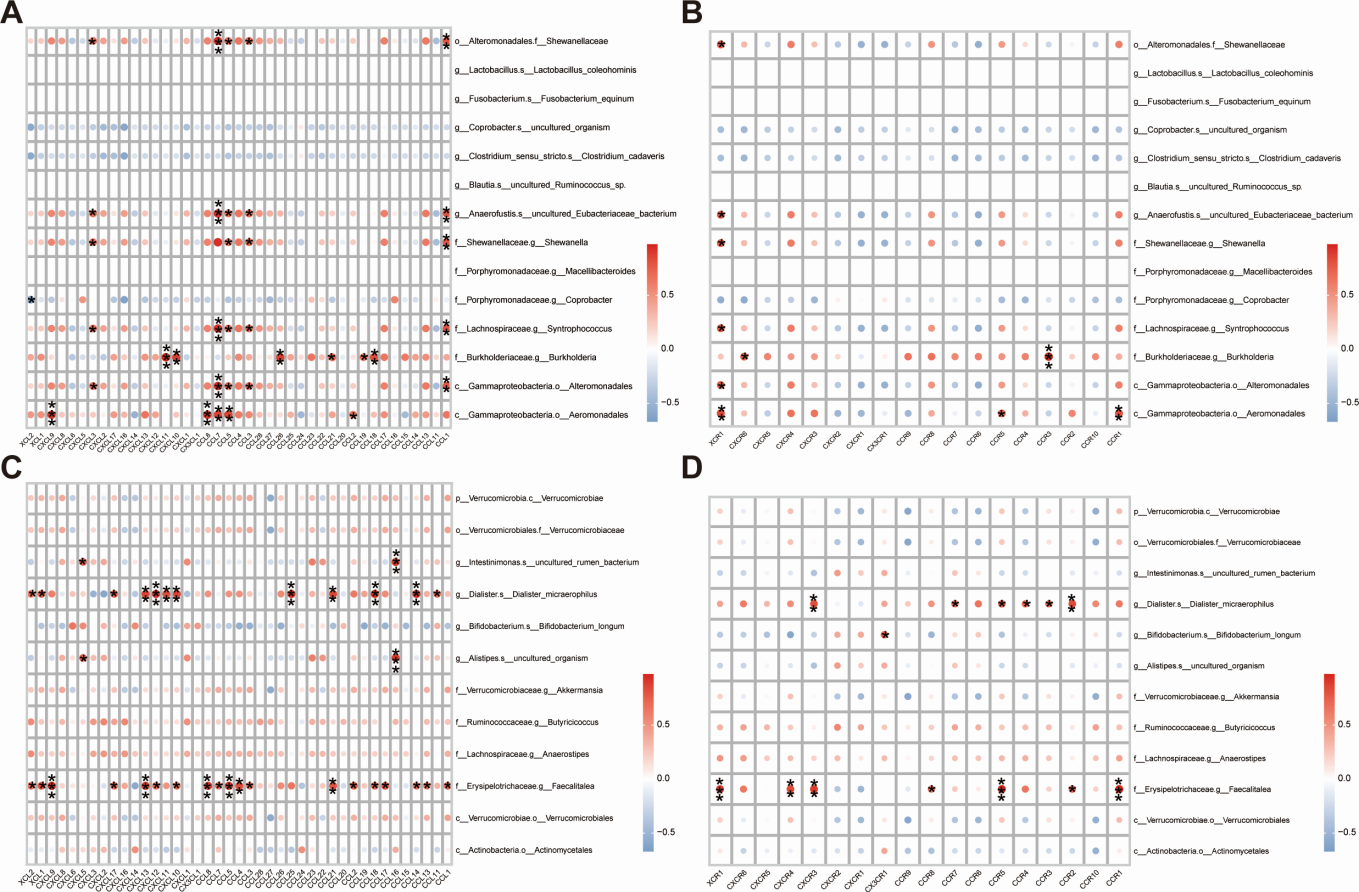
Correlation of KRAS mutation-associated gut microbiota with immune-related genes in CRC. (**A**) Heat map of the correlation between dominant gut microbiota in the KRAS mutant group and chemokines. (**B**) Heat map of the correlation between the dominant gut microbiota in the KRAS mutant group and chemokine receptors. (**C**) Heat map of the correlation between the dominant gut microbiota in the KRAS wild-type group and chemokines. (**D**) Heat map of the correlation between the dominant gut microbiota in the KRAS wild-type group and chemokine receptors. The horizontal axis of the graph represents immune-related genes, while the vertical axis represents gut microbiota. The use of red in the graph signifies a positive correlation, while blue indicates a negative correlation. The intensity of the color reflects the magnitude of Pearson’s correlation coefficient, with lighter colors indicating smaller correlation coefficient values and darker colors indicating larger values. Additionally, the presence of asterisks in the graph indicates the significance of the *P*-value: no asterisk implies a *P*-value greater than or equal to 0.05, one asterisk represents a *P*-value ranging from 0.01 to less than 0.05, two asterisks indicate a *P*-value from 0.001 to less than 0.01, and three asterisks denote a *P*-value less than 0.001.

The immune function of the human body undergoes changes during the development and growth of tumors. The immune system plays an essential role in monitoring tumor cells. To investigate the association between gut microbiota related to KRAS mutations and the immune response, we conducted an analysis of the correlation between KRAS mutation-associated gut microbiota and immune-related genes. For the dominant gut microbiota of the KRAS mutant group, *g__Anaerofustis.s__uncultured_Eubacteriaceae_bacterium*, *f__Shewanellaceae.g__Shewanella,* and *f__Lachnospiraceae.g__Syntrophococcus* showed a significant positive correlation with multiple chemokines (CXCL3, CCL5, and CCL1) and chemokine receptor (XCR1) (Fig. 6A and B; Table S4), immune-stimulating genes (CD80, CD28) (Fig. S2A; Table S4), immunosuppressive genes (CD274, HAVCR2, and PDCD1LG2) (Fig. S2B; Table S4), and immune checkpoints (LAIR1, KIR3DL1, CD86, etc.) (Fig. S2C; Table S4). For the dominant gut microbiota of the KRAS wild-type group, *f__Erysipelotrichaceae.g__Faecalitalea* and *g__Dialister.s__Dialister_micraerophilus* were notably positively associated with multiple chemokines (XCL1, CXCL10, and CCL14) and chemokine receptors (CCR2, CCR5, and CXCR3) (Fig. 6C and D; Table S4), immune-stimulating genes (CD40, KLRC1, and TNFRSF9) (Fig. S3A; Table S4), immunosuppressive genes (IDO1, LAG3, and PDCD1) (Fig. S3B; Table S4), and the immune checkpoints (CD48, ICOS, TIGIT, etc.) (Fig. S3C; Table S4). These findings emphasized the extensive connections between KRAS mutation-associated gut microbiota and immune-related genes.

### Identification of differential biological functional pathways between KRAS mutant and KRAS wild-type group and their correlation with KRAS mutation-associated gut microbiota

**Fig 7:**
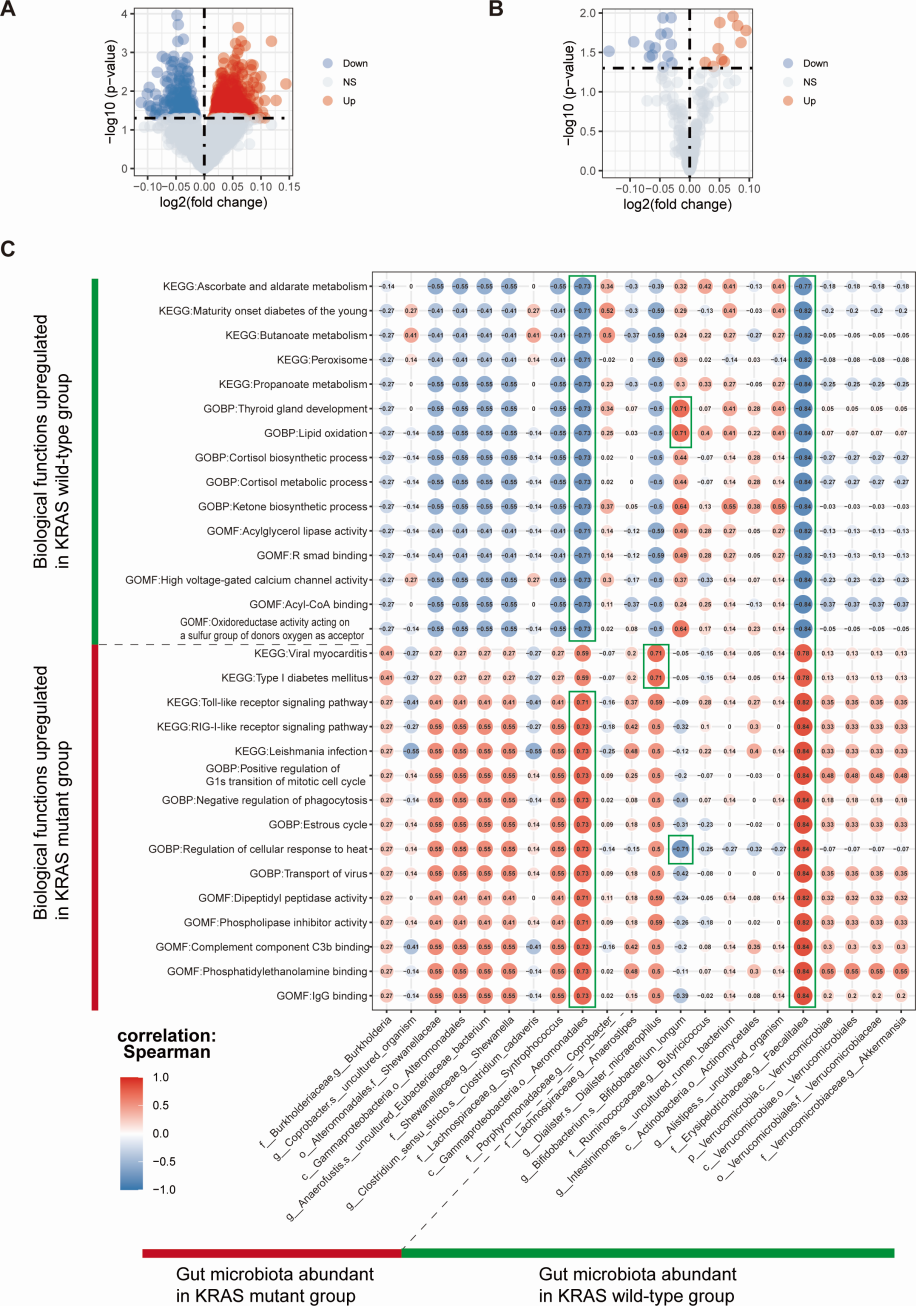
Identification of differential functional pathways between the KRAS mutant CRC group and KRAS wild-type CRC group and their correlation with KRAS mutation-associated gut microbiota in CRC. (**A**) Volcano map of colorectal KRAS mutation-associated differential GO items. (**B**) Volcano map of colorectal KRAS mutation-associated differential KEGG pathway. Each point in the graph represents the detected differentially expressed genes. Blue points indicate significantly downregulated genes, while red points indicate significantly upregulated genes. Gray points represent genes with no significant differences. The horizontal axis represents the log2FC, with points further from the central marker line indicating a greater difference multiplier. The vertical axis represents −log10 (*P*-value), and points closer to the top of the *y*-axis indicate a more significant difference in expression between the two samples. (**C**) Heat map of correlation between KRAS mutation-associated biological functions and KRAS mutation-associated gut microbiota in CRC. Sorted in ascending order of *P*-value magnitude, demonstrating the correlation between intestinal microbiota and a total of 30 biological functions. In the correlation graph, the horizontal coordinates represent KRAS mutation-associated gut microbiota, while the vertical coordinates represent mutation-associated biological functions, including GOBP items, GOMF items, and KEGG pathways. Red indicates a positive correlation, blue indicates a negative correlation, and the depth of color represents the size of Spearman’s correlation coefficient. The color intensity from light to dark corresponds to correlation coefficient values from small to large. The correlation marked by the green box indicates a *P*-value <0.05.

In pursuit of identifying differential biological regulatory pathways between the KRAS mutant and KRAS wild-type groups and investigating the correlation among these pathways and KRAS mutation-associated gut microbiota, we employed the ssGSEA method to transform RNA sequencing data from nine CRC patients into corresponding scoring matrices of GO items and KEGG pathways. In the next place, we undertook an analysis of the disparities in enriched GO terms and KEGG pathways in both groups. In the KRAS mutant group, we identified a total of 512 significantly upregulated GO items, such as GOBP:Positive regulation of monocyte chemotaxis (logFC = 0.098, *P* = 0.008) and GOMF:Complement component C3b binding (logFC = 0.107, *P* = 0.018). Moreover, 10 KEGG pathways were notably upregulated, including KEGG:Antigen processing and presentation (logFC = 0.048, *P* = 0.028) and KEGG:Cell adhesion molecules cams (logFC = 0.054, *P* = 0.039). Conversely, we found 226 GO items that were significantly upregulated in the KRAS wild-type group, such as GOBP:Innate immune response in mucosa (logFC = −0.067, *P* = 0.001) and GOBP:Organ or tissue specific immune response (logFC = −0.034, *P* = 0.015). Additionally, 14 KEGG pathways showed significant upregulation, for instance, KEGG:Base excision repair (logFC = −0.036, *P* = 0.035), as illustrated in Fig. 7A and B. Comprehensive information regarding the enrichment of GO terms and KEGG pathways can be found in Table S5. These findings collectively suggested differential biological functions between KRAS mutant and KRAS wild-type CRC.

Subsequently, we delved deeper into the relationship between the differential BP and MF items and KEGG pathways of both groups and KRAS mutation-associated gut microbiota. Notably, some of these bacteria exhibited significant correlations with these biological pathways. For instance, a substantial positive correlation was observed between *c__Gammaproteobacteria.o__Aeromonadales* and GOBP:Negative regulation of phagocytosis (*r* = 0.73, *P* = 0.025), as displayed in Fig. 7C and Table S6. Furthermore, a notable negative correlation was identified between *g__Bifidobacterium.s__Bifidobacterium_longum* and GOMF:Proteasome binding (*r* = −0.696, *P* = 0.037), as detailed in Table S6. Lastly, a significant positive correlation was observed between *f__Erysipelotrichaceae.g__Faecalitalea* and KEGG:Toll-like receptor signaling pathway (*r* = 0.822, *P* = 0.007), as depicted in Fig. 7C and Table S6. These outcomes indicated that some KRAS mutation-associated gut microbiota may influence the occurrence of KRAS mutation in CRC through distinct biological functional pathways.

### Feature tag construction of gut microbiota for KRAS mutation status prediction in CRC patients

**Fig 8:**
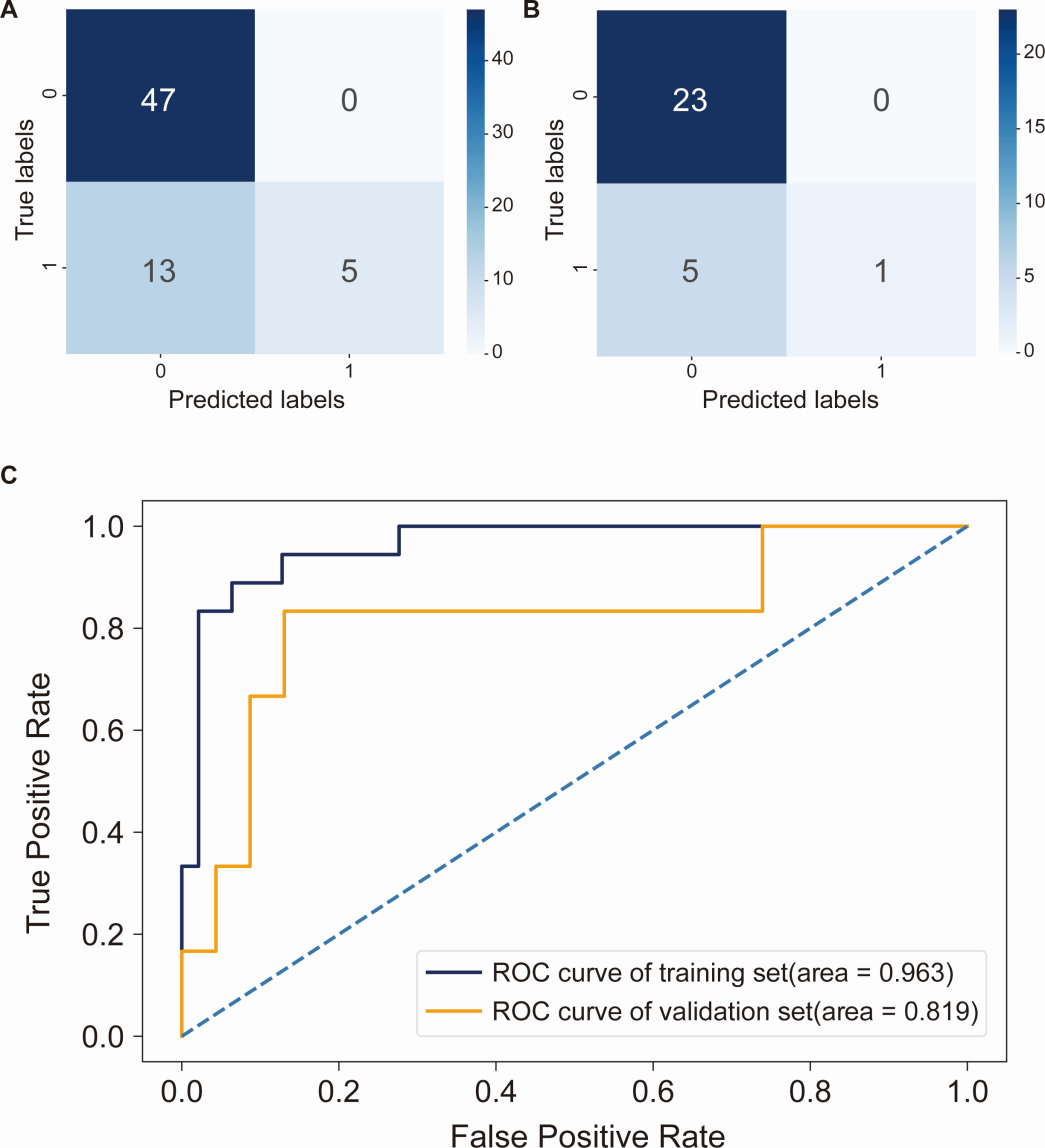
RF machine learning model for predicting KRAS mutation status in CRC based on the KRAS mutation-associated gut microbiota. (**A**) Confusion matrix in the training set of the RF model. (**B**) Confusion matrix in the validation set of RF model. The horizontal axis represents the model’s prediction label, while the vertical axis represents the true sample status. The values “1” and “0” signify positive and negative predictions, respectively. The numbers within different boxes represent the sample count. The color depth is proportional to the number of samples; the greater the number of samples, the darker the color depth. (**C**) ROC curves for the training and validation sets of the RF model. The horizontal axis displays the false positive rate of the machine learning model in predicting KRAS mutation status in CRC, while the vertical axis shows the true positive rate of this prediction. The figure’s AUC value signifies the area under the curve, with a higher AUC value indicating a more accurate model prediction.

To identify gut microbiota biomarkers associated with KRAS mutations and more accurately predict KRAS mutation status in CRC patients, we developed an RF prediction model based on KRAS mutation-associated gut microbiota. Following a careful assessment of feature importance rankings, we retained 73% of the 26 KRAS mutation-associated gut microbiota features, namely, the top 19 KRAS mutation-associated gut microbiota, for subsequent inclusion in the RF model construction (as depicted in Fig. S4). Upon analyzing the confusion matrix of the training set for the RF prediction model, we observed that the number of samples predicted as true negatives (TN) significantly exceeded that of false negatives (FN), and the number of samples predicted as true positives (TP) notably overpassed that of false positives (FP). However, the number of samples predicted as FN was marginally higher than that of TP (refer to Fig. 8A). A similar trend was seen in the confusion matrix of the validation set (see Fig. 8B). The ROC curve based on the RF model revealed that the AUC value for the training set was 0.963, and for the validation set, it stood at 0.819 (see Fig. 8C). In summary, although the RF-based KRAS prediction model exhibited a modest false negative rate, the ROC curves demonstrated promising predictive efficiency for both the training set (AUC > 0.9) and the validation set (AUC > 0.8). This suggested that the RF model held significant potential in predicting KRAS mutation status in CRC patients with a reliable degree of accuracy, thus offering a novel strategy for the precise treatment of CRC.

## DISCUSSION

To the best of our knowledge, this study represents the first attempt to investigate the correlation between gut microbiota and KRAS mutation in CRC. Within this study, we conducted 16S rRNA sequencing on stool samples obtained from KRAS mutant and KRAS wild-type CRC patients. Our objective was to identify gut microbiota associated with KRAS mutation and explore potential biological pathways linked to them. Additionally, we aimed to develop microbial markers for predicting and identifying KRAS mutation status in CRC patients. Through an analysis of the richness and diversity of gut microbiota in both groups, we observed that the KRAS mutant group exhibited higher values for observed OTUs, Chao1 index, ACE index, and Shannon index when compared to the KRAS wild-type group. Notably, the Simpson index was lower in the KRAS mutant group, though this difference did not reach statistical significance (as shown in Fig. 2A and B). This lack of significance may be attributed to the limited sample size. At present, no literature has addressed the diversity of gut microbiota in relation to KRAS mutation status in CRC. Consequently, we posited that KRAS mutation might be associated with increased abundance and diversity of gut microbiota in CRC patients. Further expansion of our sample size is essential to confirm this hypothesis. Utilizing PLS-DA, we discerned that CRC patients with distinct KRAS mutation status could be distinguished by their gut microbiota composition. This observation indicated a discernible disparity in the composition of gut microbiota between the two groups.

We ascertained 26 distinct types of gut microbiota with significance between the KRAS mutant and KRAS wild-type groups by LEfSe analysis. Specifically, at the genus level, *Fusobacterium*, *Clostridium*, and *Shewanella* were all abundant in the KRAS mutant group. Notably, these three genera ranked prominently in our RF model in terms of feature importance among the gut microbiota associated with KRAS mutations (refer to Fig. S4). *Fusobacterium*, classified as a Gram-negative anaerobic bacillus, is an opportunistic pathogen found in both the oral cavity and gastrointestinal tract (37). It is worth mentioning that recent studies have established a strong link between *Fusobacterium* and CRC development (38). Kim and colleagues (39) observed a significant increase in *Fusobacterium* abundance in CRC compared to adjacent normal tissues. Additionally, in the early stage of CRC, the virulence factor of *Fusobacterium-Fusobacterium* adhesin A can selectively bind to E-cadherin, subsequently activating the β-catenin signaling pathway, which in turn induces inflammatory responses and carcinogenesis (10). Meanwhile, Huangfu and their team (40) noted that elevated levels of *Fusobacterium nucleatum* were not only closely associated with KRAS mutation but also correlated with chemoresistance in CRC, aligning with our own findings. *Clostridium*, associated with CRC, is able to produce metabolites (such as ammonia, hydrogen sulfide, indoles, and phenols) inside the large intestine. These metabolites are known to cause DNA damage and trigger inflammatory responses, thereby increasing the risk of CRC development (41). Our study found a significant enrichment of *Clostridium* in the KRAS mutant group, leading us to hypothesize a potential correlation between *Clostridium* and KRAS mutation in CRC patients. Similarly, *Shewanella* has been proved to be a contributor to CRC development. In the early stages of CRC, the abundance of *Shewanella* was relatively reduced. However, as CRC progressed, *Shewanella* took advantage of the tumor’s metabolism, leading to competitive proliferation on the tumor’s surface, ultimately promoting CRC development (38). In light of these findings, it is plausible to consider *Fusobacterium*, *Clostridium*, and *Shewanella* as potential biomarkers for identifying KRAS mutation in CRC patients.

Compared with the KRAS mutant group, the primary dominant gut microbiota in the KRAS wild-type group consisted of *Bifidobacterium* and *Akkermansia. Bifidobacterium* is a probiotic with antitumor activity, capable of producing glutathione, thereby reducing DNA damage (42). Shi et al. (43) confirmed that *Bifidobacterium* exerted antitumor effects on a mouse model by obstructing the signal emitted by CD47 on tumor cells, thus enhancing macrophage phagocytosis. It has been reported that there was an inverse correlation between the development of KRAS mutation and the abundance of *Bifidobacterium* in CRC (44). These previous studies were in agreement with our findings. In addition, we observed a higher abundance of *Akkermansia* in the KRAS wild-type group. *Akkermansia* is a Gram-negative anaerobic bacterium that is widespread in the human gut and currently recognized as a potential probiotic (45). It has been demonstrated (46) that *Akkermansia* can downregulate pro-inflammatory factors such as tumor necrosis factor (TNF-α), interleukin 6 (IL-6), and monocyte chemoattractant protein 1 in colitis tissues, contributing to enhanced colonic epithelial barrier and relief from colonic inflammatory responses, which are attributed to *Akkermansia*’s role in NCRP3 activation for the subsequent release of IL-1b and IL-18. In short, we came to the speculation that CRC patients may have a reduced likelihood of developing KRAS mutation in the presence of some gut microbiota such as *Bifidobacterium* and *Akkermansia*, which, to some extent, hinder the progression of CRC.

We utilized PICRUSt2 software to predict differential KEGG pathways between the gut microbiota of KRAS mutant and KRAS wild-type CRC patients, with the aim of identifying enriched biological pathways of gut microbiota associated with KRAS mutation status in CRC. The abundance of the isoflavonoid biosynthesis pathway was found to be significantly higher in the KRAS wild-type group compared to the KRAS mutant group. Isoflavones represent a group of secondary metabolites originating from the phenylpropanoid pathway (47). Genistein is a major isoflavone derived from soybeans. A study by Qin et al. (48) revealed that genistein inhibited the phosphorylation of Akt in human CRC cells (HCT-116 cells) by downregulating the mRNA expression of Akt, SGK1, and miR-95, thereby hindering CRC development. Concurrently, research by Zhang et al. (49) demonstrated that genistein-treated tumor-transplanted nude mice exhibited reduced tumor growth, coupled with a dose-dependent downregulation of MCL1, APP, and KDR protein and mRNA. Additionally, calycosin, a significant component of isoflavones, has been shown to have the potential to suppress CRC through the ERβ-mediated regulation of miR-17 and PTEN expression (50). Overall, in comparison to KRAS mutant CRC, it is postulated that KRAS wild-type CRC may be less aggressive due to the upregulation of the isoflavonoid biosynthesis pathway, which may inhibit CRC development and progression.

In order to explore the relationship between gut microbiota and genetic factors in CRC, we conducted an analysis of the correlation among KRAS mutation-associated gut microbiota, TIICs, immune-related genes, and biological pathways. Notably, a significant positive correlation emerged between *Bifidobacterium* and mast cells (MCs), as illustrated in Fig. 5C. Furthermore, *Bifidobacterium longum* exhibited a substantial positive correlation with the chemokine receptor CX3CR1, as evidenced by Fig. 6D. Interestingly, we observed a higher abundance of *Bifidobacterium longum* in the KRAS wild-type group compared to the KRAS mutant group, suggesting a negative association between *Bifidobacterium longum* and KRAS mutation occurrence. Importantly, *Bifidobacterium* was found to be less abundant in CRC patients in comparison to their healthy counterparts (51). CX3CR1 represents a classical G protein-coupled receptor that can be expressed on a subset of MCs (52). Several studies have highlighted the role of MCs in tumor development, primarily through the release of granulosa trypsin, which promotes angiogenesis (53). Therefore, *Bifidobacterium* may activate MCs by upregulating CX3CR1 expression, which may lead to the suppression of CRC development. We also observed a significant negative correlation between *Bifidobacterium* and GOMF:Proteasome binding (*r* = −0.696, *P* = 0.037, Table S6). Proteasomes are mega-protein complexes that are ubiquitously present in the cytoplasm and nucleus of all eukaryotic cells, which are mainly responsible for the degradation of superfluous proteins (54). In mammalian proteasomes, the serine protease plays a crucial role (55). Serpin, belonging to the serine protease inhibitor superfamily, acts as an antagonist of serine protease activity (56) and plays a regulatory role in essential pathways of coagulation, immunity, and even cancer in humans (57). Serpin, when isolated from *Bifidobacterium*, has been strongly associated with intestinal inflammation. Studies have demonstrated that *Bifidobacterium* can release neutrophil elastase (NE) inhibitors at the site of intestinal inflammation (58). By inhibiting NE activity and reducing the production of pro-inflammatory cytokines such as TNF-α and IL-6, *Bifidobacterium* plays a crucial role in suppressing the intestinal inflammatory response and safeguarding the intestinal mucosa (58). Thus, *Bifidobacterium* may play a role in inhibiting the development and propagation of CRC by the secretion and release of anti-inflammatory substances that prevent the pro-inflammatory effect of proteasome. However, the above assumptions were based on correlation analysis and previous literature results; further experimentation is required to confirm these hypotheses.

In pursuit of fecal biomarkers associated with KRAS mutation in CRC, we integrated KRAS mutation-associated gut microbiota into the construction of a RF predictive model. While the confusion matrix of both the training and validation sets in the RF prediction model for KRAS mutation status displayed a marginally higher false negative rate (refer to Fig. 8A and B), it is noteworthy that the ROC curve of this RF model for forecasting KRAS mutation status in CRC patients exhibited an AUC value exceeding 0.9 in the training set and an AUC value surpassing 0.8 in the validation set (as shown in Fig. 8C), which underscored the model’s substantial predictive efficacy. These findings strongly implied that the RF predictive model held considerable potential for clinical application, offering a novel dimension in the prediction of KRAS mutation status among CRC patients in a clinical setting.

There were limitations in this study. Firstly, the KRAS mutation status of five CRC patients was predicted using the CC method, which may introduce bias compared to the actual situation due to the limited size of the sample with transcriptome sequencing data. Nevertheless, this approach was based on the KRAS mutation-associated DEGs matrix and the PCA showed obvious clustering, which still held some validity. Hence, it would be advisable to incorporate a more extensive data set of CRC patients with KRAS phenotype and transcriptome sequencing data into future large-scale investigations. Secondly, given that this is a single-center study, it is imperative to corroborate our findings through multicenter studies with larger sample sizes. Additionally, in contrast to metagenomic sequencing, 16S rRNA sequencing lacks the depth required to identify specific bacteria at the species level (59). Metagenomic sequencing, in contrast, enables high-throughput sequencing of all microbial genomes in each sample and comprehensive species-level annotation (60). Therefore, the inclusion of metagenomic sequencing data for additional support is often recommended. Furthermore, it is crucial to acknowledge that fecal samples may not provide a complete representation of the entire gut microbiota landscape; thus, consideration of additional endoscopic sampling of gut mucosal samples for co-analysis is warranted to obtain a more comprehensive data set of microbial information. Last but not least, it is worth noting that metabolomics analysis, widely employed across various research domains, has the capability to detect subtle variations in biological metabolic pathways, which plays a pivotal role in further exploring potential microbial functions (61). Thus, including metabolomics analysis in future research endeavors is advisable.

### Conclusion

In our study, we identified 26 KRAS mutation-associated gut microbiota in CRC patients. *Bifidobacterium* may play a crucial role in inhibiting the growth and development of CRC, which might be associated with the upregulation of MCs and CX3CR1 expression, along with the downregulation of the GOMF:Proteasome binding. Furthermore, the RF model, developed on the basis of KRAS mutation-associated gut microbiota, holds significant potential for clinical application in predicting KRAS mutation status among CRC patients.

## Data Availability

The original contributions presented in the study are included in the article material; further inquiries can be directed to the corresponding authors. The data that support the findings of this study are openly available in the National Genomics Data Center (NGDC) database at https://www.cncb.ac.cn/, accession number: HRA006887.

## SUPPLEMENTAL MATERIAL

Fig. S1 spectrum.02720-23-s0001.docx Consistent clustering analysis on the basis of KRAS mutation-associated differentially expressed gene matrix in CRC.

Fig. S2 spectrum.02720-23-s0002.docx Correlation of dominant gut microbiota with immune activation genes, immune suppression genes, and checkpoints in KRAS mutant groups.

Fig. S3 spectrum.02720-23-s0003.docx Correlation of dominant gut microbiota with immune activating genes, immune suppressor genes, and checkpoints in KRAS wild-type group.

Fig. S4 spectrum.02720-23-s0004.docx Ranking the importance of KRAS mutation-associated gut microbiota variates for predicting KRAS phenotype for CRC patients.

Table S1 spectrum.02720-23-s0005.xlsx KRAS mutation-associated differentially expressed genes.

Table S2 spectrum.02720-23-s0006.docx Results of LEfSe analysis of gut microbiota between KRAS mutant and KRAS wild-type CRC patients.

Table S3 spectrum.02720-23-s0007.xlsx Functional KEGG pathways of the differential gut microbiota between KRAS mutant and KRAS wild-type CRC patients.

Table S4 spectrum.02720-23-s0008.xlsx Correlation matrix among immune-related genes and KRAS mutation-associated gut microbiota.

Table S5 spectrum.02720-23-s0009.xlsx Differential biological functions between KRAS mutant and KRAS wild-type CRC patients.

Table S6 spectrum.02720-23-s0010.xlsx Significant correlation matrix among enriched biological functions and KRAS mutation-associated gut microbiota.

ASM does not own the copyrights to Supplemental Material that may be linked to, or accessed through, an article. The authors have granted ASM a non-exclusive, world-wide license to publish the Supplemental Material files. Please contact the corresponding author directly for reuse.
