# Supplementary material for: Identification of KRAS mutation-associated gut microbiota in colorectal cancer and construction of predictive machine learning model
Source: Microbiol Spectr. 2024 Apr 4;12(5):e02720-23. doi: 10.1128/spectrum.02720-23 (PMC11064510; doi:10.1128/spectrum.02720-23)
Supplement: Fig. S1 — Consistent clustering analysis on the basis of KRAS mutation-associated differentially expressed gene matrix in CRC. [file spectrum.02720-23-s0001.docx]

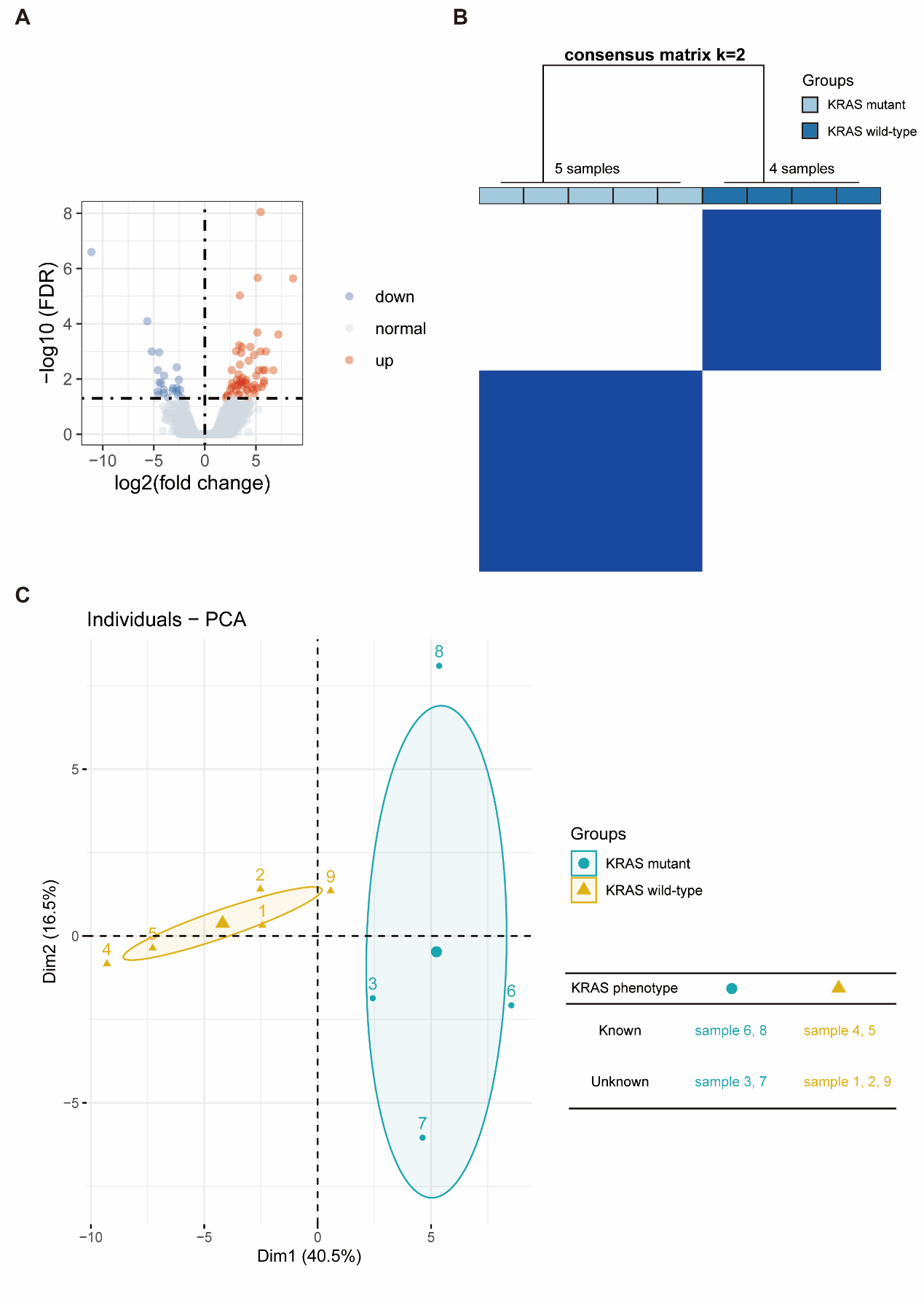


**Supplementary Figure**

**Supplementary Figure 1. Consistent clustering analysis on the basis of KRAS mutation-associated differentially expressed gene matrix in CRC**

**(A) Volcano map of colorectal KRAS mutation-associated differentially expressed genes.** Each point in the graph represents the detected differentially expressed genes. Blue points indicate significantly down-regulated genes, while red points indicate significantly up-regulated genes. Gray points represent genes with no significant differences. The horizontal axis represents the log2 fold change (log2FC), with points further from the central marker line indicating a greater difference multiplier. The vertical axis represents -log10 (FDR), and points closer to the top of the y-axis indicate a more significant difference in expression between the two samples. **(B) Consensus matrix of 9 CRC patients (k=2).** The different colors represent the different subgroups of the identified clusters. **(C) PCA of 9 patients with CRC.** The horizontal axis (Dim1) and the vertical axis (Dim2) represent the two primary components that offer the most comprehensive explanation for differences among samples. The scale denotes relative distances. Each point in the graph represents a sample, with varying colors indicating the respective group to which the sample belongs. Closely clustered points indicate greater sample similarity.
