# Supplementary material for: Identification of KRAS mutation-associated gut microbiota in colorectal cancer and construction of predictive machine learning model
Source: Microbiol Spectr. 2024 Apr 4;12(5):e02720-23. doi: 10.1128/spectrum.02720-23 (PMC11064510; doi:10.1128/spectrum.02720-23)
Supplement: Fig. S2 — Correlation of dominant gut microbiota with immune activation genes, immune suppression genes, and checkpoints in KRAS mutant groups. [file spectrum.02720-23-s0002.docx]

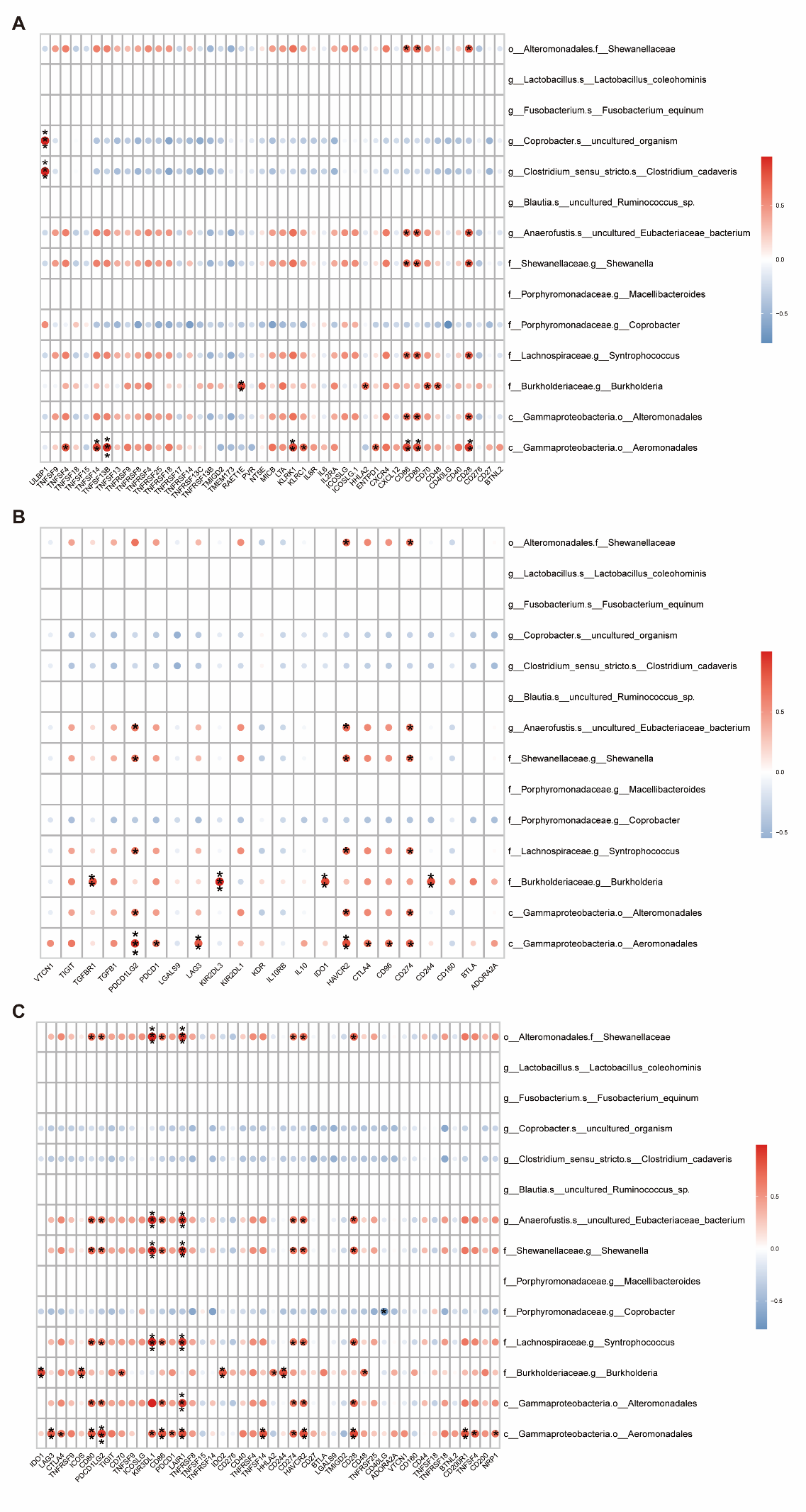


**Supplementary Figure 2. Correlation of dominant gut microbiota with immune activation genes, immune suppression genes and checkpoints in KRAS mutant groups**

**(A) Heat map of the correlation between the dominant gut microbiota in KRAS mutant group and immune activation genes. (B) Heat map of the correlation between the dominant** **gut microbiota in the KRAS mutant group and immunosuppressive genes. (C) Heat map of correlation between the dominant gut microbiota in KRAS mutant group and checkpoints.** The horizontal axis of the graph represents immune-related genes, while the vertical axis represents gut microbiota. The use of red in the graph signifies a positive correlation, while blue indicates a negative correlation. The intensity of the color corresponds to the magnitude of Pearson's correlation coefficient, with lighter shades representing smaller correlation coefficients and darker shades representing larger coefficients. Additionally, the presence of asterisk (*) in the graph indicates the significance of the P-value: no asterisk (*) implies a P-value greater than or equal to 0.05, one asterisk (*) represents a P-value ranging from 0.01 to less than 0.05, two asterisks (**) indicate a P-value from 0.001 to less than 0.01, and three asterisks (***) denote a P-value less than 0.001.
