# Supplementary material for: Identification of KRAS mutation-associated gut microbiota in colorectal cancer and construction of predictive machine learning model
Source: Microbiol Spectr. 2024 Apr 4;12(5):e02720-23. doi: 10.1128/spectrum.02720-23 (PMC11064510; doi:10.1128/spectrum.02720-23)
Supplement: Fig. S3 — Correlation of dominant gut microbiota with immune activating genes, immune suppressor genes, and checkpoints in KRAS wild-type group. [file spectrum.02720-23-s0003.docx]

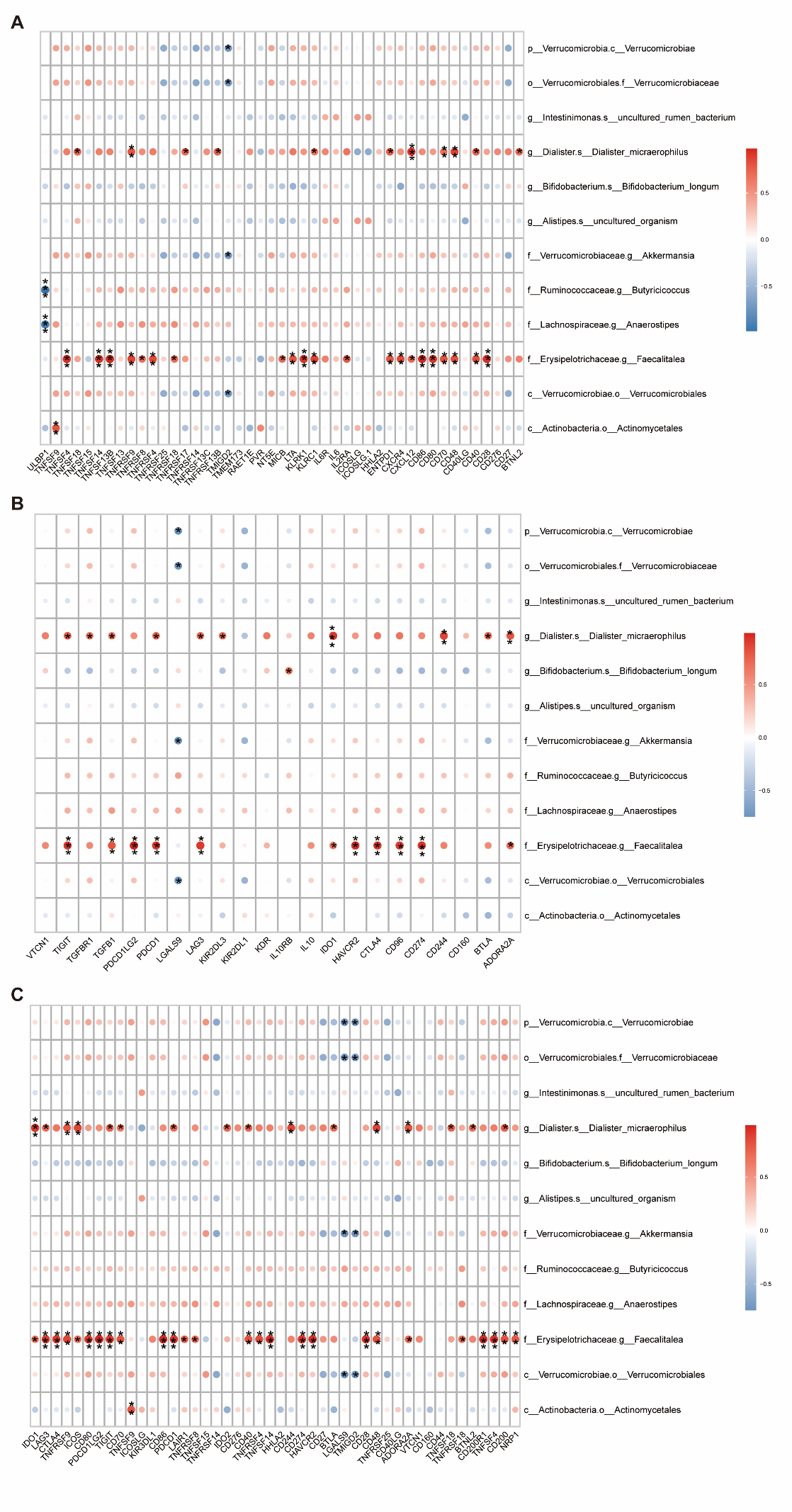


**Supplementary Figure 3. Correlation of dominant gut microbiota with immune activating genes, immune suppressor genes and checkpoints in KRAS wild-type group**

**(A) Heat map of the correlation between the dominant gut microbiota** **in the KRAS wild-type group and immune activation genes. (B) Heat map of the correlation between dominant gut microbiota in the KRAS wild-type group and immunosuppressive genes. (C) Heat map of correlation between dominant gut microbiota in the KRAS wild-type group and checkpoints.** The horizontal axis represents immune-related genes, while the vertical axis represents bacteria. The red color within the graph signifies a positive correlation, whereas the blue color indicates a negative correlation. The depth of the color represents the magnitude of Pearson's correlation coefficient, with darker shades indicating stronger correlation coefficients. Additionally, the presence of asterisk (*) in the graph indicates the significance of the P-value: no asterisk (*) implies a P-value greater than or equal to 0.05, one asterisk (*) represents a P-value ranging from 0.01 to less than 0.05, two asterisks (**) indicate a P-value from 0.001 to less than 0.01, and three asterisks (***) denote a P-value less than 0.001.
