# Supplementary material for: Identification of KRAS mutation-associated gut microbiota in colorectal cancer and construction of predictive machine learning model
Source: Microbiol Spectr. 2024 Apr 4;12(5):e02720-23. doi: 10.1128/spectrum.02720-23 (PMC11064510; doi:10.1128/spectrum.02720-23)
Supplement: Fig. S4 — Ranking the importance of KRAS mutation-associated gut microbiota variates for predicting KRAS phenotype for CRC patients. [file spectrum.02720-23-s0004.docx]

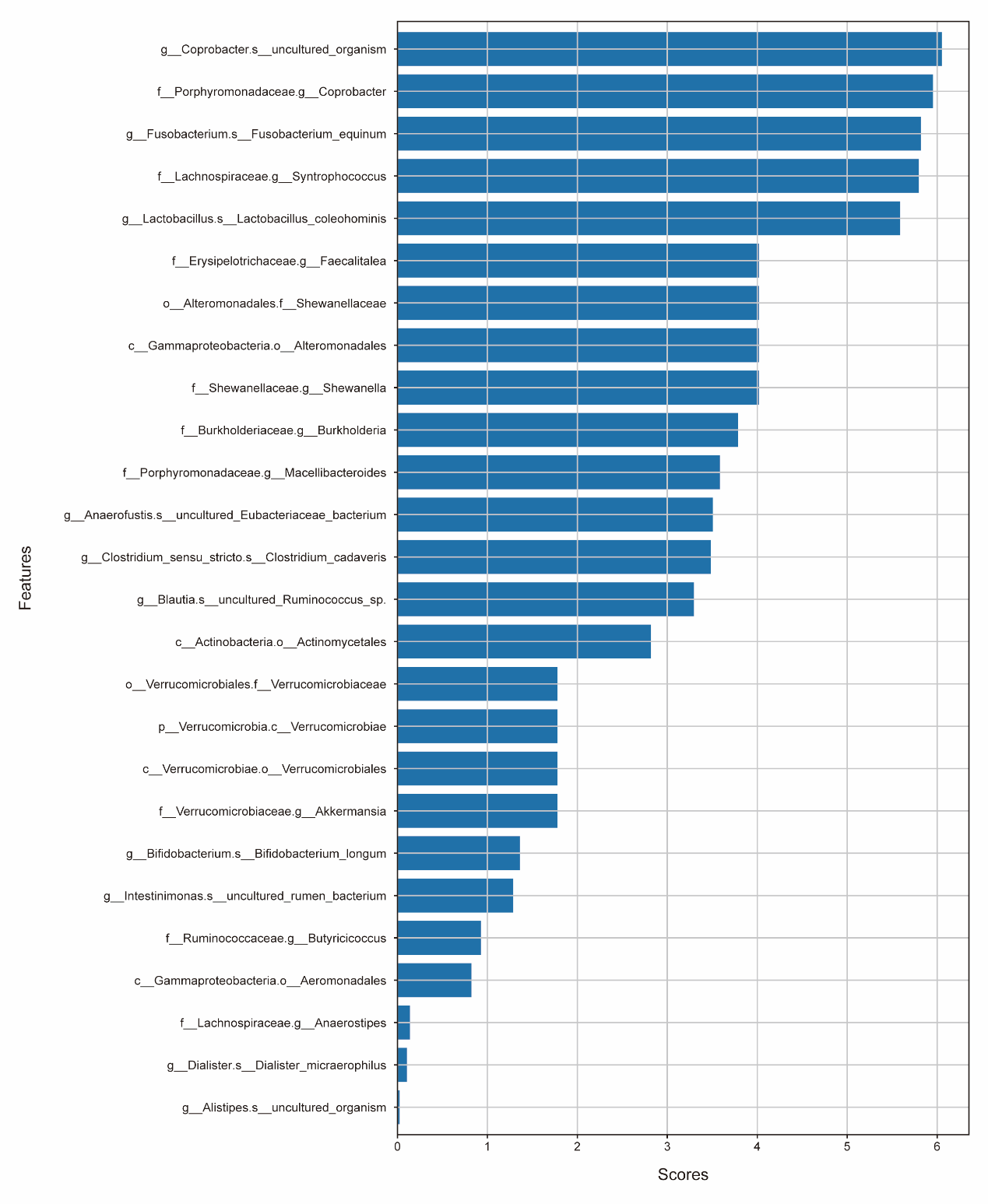


**Supplementary Figure 4.** **Ranking the importance of KRAS mutation-associated gut microbiota variates for predicting KRAS phenotype for CRC patients.** The vertical axis represents the KRAS mutation-associated gut microbiota, which function as included features in the Random Forest (RF) model. The horizontal axis of the graph displays a score that quantifies the feature's importance; the higher the score, the greater the significance of the feature.
