# Supplementary material for: Identification of KRAS mutation-associated gut microbiota in colorectal cancer and construction of predictive machine learning model
Source: Microbiol Spectr. 2024 Apr 4;12(5):e02720-23. doi: 10.1128/spectrum.02720-23 (PMC11064510; doi:10.1128/spectrum.02720-23)
Supplement: Table S2 — Results of LEfSe analysis of gut microbiota between KRAS mutant and KRAS wild-type CRC patients. [file spectrum.02720-23-s0006.docx]

**Supplementary Table 2. Results of LEfSe analysis of gut microbiota between KRAS mutant and KRAS wild-type CRC patients**

| Taxonomy | Group | LDA (log10) | P value |
| --- | --- | --- | --- |
| *f__Porphyromonadaceae.g__Macellibacteroides* | KRAS mutant | 4.310236 | 0.015175 |
| *g__Anaerofustis.s__uncultured_Eubacteriaceae_bacterium* | KRAS mutant | 4.285 | 0.015175 |
| *o__Alteromonadales.f__Shewanellaceae* | KRAS mutant | 4.198308 | 0.015175 |
| *c__Gammaproteobacteria.o__Alteromonadales* | KRAS mutant | 4.197389 | 0.015175 |
| *g__Blautia.s__uncultured_Ruminococcus_sp_* | KRAS mutant | 4.186996 | 0.015175 |
| *f__Shewanellaceae.g__Shewanella* | KRAS mutant | 4.179482 | 0.015175 |
| *f__Burkholderiaceae.g__Burkholderia* | KRAS mutant | 4.121415 | 0.002791 |
| *g__Fusobacterium.s__Fusobacterium_equinum* | KRAS mutant | 4.033345 | 0.015175 |
| *g__Coprobacter.s__uncultured_organism* | KRAS mutant | 3.966182 | 0.002791 |
| *g__Lactobacillus.s__Lactobacillus_coleohominis* | KRAS mutant | 3.592089 | 0.015175 |
| *f__Porphyromonadaceae.g__Coprobacter* | KRAS mutant | 3.404538 | 0.047575 |
| *f__Lachnospiraceae.g__Syntrophococcus* | KRAS mutant | 3.366653 | 0.021825 |
| *g__Clostridium_sensu_stricto. s__Clostridium_cadaveris* | KRAS mutant | 3.313731 | 0.021825 |
| *c__Gammaproteobacteria.o__Aeromonadales* | KRAS mutant | 2.41754 | 0.046703 |
| *g__Bifidobacterium.s__Bifidobacterium_longum* | KRAS wild-type | 5.441929 | 0.022502 |
| *p__Verrucomicrobia.c__Verrucomicrobiae* | KRAS wild-type | 5.40511 | 0.049482 |
| *c__Verrucomicrobiae.o__Verrucomicrobiales* | KRAS wild-type | 5.40511 | 0.049482 |
| *o__Verrucomicrobiales.f__Verrucomicrobiaceae* | KRAS wild-type | 5.40511 | 0.049482 |
| *f__Verrucomicrobiaceae.g__Akkermansia* | KRAS wild-type | 5.40511 | 0.049482 |
| *f__Lachnospiraceae.g__Anaerostipes* | KRAS wild-type | 4.783522 | 0.007867 |
| *c__Actinobacteria.o__Actinomycetales* | KRAS wild-type | 3.89914 | 0.043178 |
| *f__Ruminococcaceae.g__Butyricicoccus* | KRAS wild-type | 3.88325 | 0.035495 |
| *g__Alistipes.s__uncultured_organism* | KRAS wild-type | 3.789014 | 0.045239 |
| *g__Intestinimonas.s__uncultured_rumen_bacterium* | KRAS wild-type | 3.654102 | 0.036471 |
| *g__Dialister.s__Dialister_micraerophilus* | KRAS wild-type | 3.516604 | 0.020753 |
| *f__Erysipelotrichaceae.g__Faecalitalea* | KRAS wild-type | 3.128672 | 0.046703 |

Footnote: Taxonomy: information of KRAS mutation-associated gut microbiota; Group: KRAS mutant CRC and KRAS wild-type CRC patients; LDA (log10): effect value of KRAS mutation-associated gut microbiota, which was after log10 transformation; the species showed in the table were selected by the threshold of |LDA score| >2 and P < 0.05. P value: P<0.05 as statistical significance.
